# DNA demethylases target promoter transposable elements to positively regulate stress responsive genes in *Arabidopsis*

**DOI:** 10.1186/s13059-014-0458-3

**Published:** 2014-09-17

**Authors:** Tuan-Ngoc Le, Ulrike Schumann, Neil A Smith, Sameer Tiwari, Phil Chi Khang Au, Qian-Hao Zhu, Jennifer M Taylor, Kemal Kazan, Danny J Llewellyn, Ren Zhang, Elizabeth S Dennis, Ming-Bo Wang

**Affiliations:** CSIRO Division of Plant Industry, Clunies Ross Street, Canberra, ACT 2061 Australia; CSIRO Division of Plant Industry, 306 Carmody Road, St Lucia, QLD 4067 Australia; School of Biological Sciences, University of Wollongong, Northfields Avenue, Wollongong, NSW 2522 Australia

## Abstract

**Background:**

DNA demethylases regulate DNA methylation levels in eukaryotes. *Arabidopsis* encodes four DNA demethylases, *DEMETER* (*DME*), *REPRESSOR OF SILENCING 1* (*ROS1*), *DEMETER-LIKE 2* (*DML2*), and *DML3*. While *DME* is involved in maternal specific gene expression during seed development, the biological function of the remaining DNA demethylases remains unclear.

**Results:**

We show that ROS1, DML2, and DML3 play a role in fungal disease resistance in *Arabidopsis*. A triple DNA demethylase mutant, *rdd* (*ros1 dml2 dml3*), shows increased susceptibility to the fungal pathogen *Fusarium oxysporum*. We identify 348 genes differentially expressed in *rdd* relative to wild type, and a significant proportion of these genes are downregulated in *rdd* and have functions in stress response, suggesting that DNA demethylases maintain or positively regulate the expression of stress response genes required for *F. oxysporum* resistance. The *rdd*-downregulated stress response genes are enriched for short transposable element sequences in their promoters. Many of these transposable elements and their surrounding sequences show localized DNA methylation changes in *rdd*, and a general reduction in CHH methylation, suggesting that RNA-directed DNA methylation (RdDM), responsible for CHH methylation, may participate in DNA demethylase-mediated regulation of stress response genes. Many of the *rdd*-downregulated stress response genes are downregulated in the RdDM mutants *nrpd1* and *nrpe1*, and the RdDM mutants *nrpe1* and *ago4* show enhanced susceptibility to *F. oxysporum* infection.

**Conclusions:**

Our results suggest that a primary function of DNA demethylases in plants is to regulate the expression of stress response genes by targeting promoter transposable element sequences.

**Electronic supplementary material:**

The online version of this article (doi:10.1186/s13059-014-0458-3) contains supplementary material, which is available to authorized users.

## Background

DNA cytosine methylation is one of the main epigenetic mechanisms in higher eukaryotes, and plays a key role in maintaining genome stability and regulating gene expression. In plants, cytosine methylation levels are controlled by multiple pathways, including *de novo* methylation, maintenance methylation, and demethylation [1]. *De novo* cytosine methylation is mediated by RNA-directed DNA methylation (RdDM), a plant-specific pathway that can generate 5-methylcytosines at all sequence contexts (CG, CHG, and CHH where H stands for A, C, or T) [2]. RdDM is directed by 24-nt small interfering RNAs (siRNAs) produced by the combined function of RNA POLYMERASE IV (Pol IV), RNA-DEPENDENT RNA POLYMERASE 2 (RDR2), and DICER-LIKE 3 (DCL3). These siRNAs bind to ARGONAUTE 4 (AGO4) to form and guide the RNA-induced silencing complex to target DNA through interaction with long non-coding RNA transcribed by Pol V. This AGO4-siRNA-long non-coding RNA complex then recruits the *de novo* methyltransferase DRM2 (and DRM1) *via* an unknown mechanism, resulting in sequence-specific cytosine methylation. The symmetric CG and CHG methylation, once formed, can be maintained during DNA replication by the methyltransferases MET1 (for CG methylation) and CMT3 (for CHG methylation). However, CHH methylation does not persist during DNA replication and must be generated *de novo* by the 24-nt siRNA-directed RdDM pathway. In plants DNA methylation occurs mainly in transposons and repetitive DNA sequences [1].

Plants, like mammals, possess an active DNA demethylation process catalyzed by the DNA glycosylase family of DNA demethylases [3,4]. Four DNA demethylases, namely DEMETER (DME), REPRESSOR OF SILENCING 1 (ROS1)/DEMETER-LIKE 1 (DML1), DML2, and DML3, have been identified in Arabidopsis. These DNA glycosylase enzymes remove 5-methylcytosine and replace it with an unmethylated cytosine through a base excision repair mechanism [3]. *DME* is expressed primarily in the central cell of the female gametophyte and is required for the maternal allele-specific expression of imprinted genes in the central cell and endosperm [4]. The other three demethylases in Arabidopsis are thought to account for all demethylase activity in somatic tissues, but their biological functions are poorly understood. Of the three demethylases, *ROS1* is the most highly expressed and has been shown to repress transcriptional silencing of transgenes and endogenous genes [5]. The Arabidopsis *ros1* or *ros1 dml2 dml3* (*rdd*) mutants show no obvious developmental defects under normal growth conditions [6], and only a small number (hundreds) of genomic loci in the *rdd* mutant show changes in DNA methylation or gene expression [6,7].

Recent studies have suggested that DNA methylation plays an important role in plant stress responses. For instance, exposure to biotic stress such as pathogen attack leads to a dynamic methylation changes across the Arabidopsis genome [8]. The RdDM mutant *ago4* has increased susceptibility to infection with the bacterial pathogen *Pseudomonas syringae* [9], whereas the *polV* mutant shows enhanced resistance to this pathogen [10]. Like the *polV* mutant, the methylation-deficient mutants *met1* and *ddc* (*drm1 drm2 cmt3*) show enhanced resistance to *P. syringae* [8], raising the possibility that DNA demethylation plays a positive role in plant disease resistance. Consistent with this, resistance to *P. syringae* [11] or response to bacterial flagellin [12] is correlated with overall hypomethylation of DNA in Arabidopsis. Furthermore, the *ros1* mutant shows increased susceptibility to *P. syringae* and this coincides with enhanced cytosine methylation in a transposon inserted into a disease resistance gene promoter compromising the expression of this gene in *ros1* [12].

In contrast to bacterial pathogens, few studies have examined the role of epigenetic pathways in plant defence against fungal pathogens [10,13]. In this study we have investigated potential roles of epigenetic mechanisms in plant disease resistance using the fungal pathogen, *Fusarium oxysporum. F. oxysporum* is a root-infecting, hemi-biotrophic fungal pathogen that gains entry into the host plant through lateral roots and subsequently spreads to the aerial parts of the plant. *F. oxysporum* infects a large variety of plant species including important crop plants such as tomato, melon, bean, cotton, and banana. *F. oxysporum* f. sp. *conglutinans* (*Fo*) strain 5176 used in this study infects *Arabidopsis thaliana* and causes distinct leaf chlorosis and often plant death. We found that the triple DNA demethylase mutant, *rdd*, shows enhanced susceptibility to *Fo* infection. In addition, we show that the loss of function of the three DNA demethylases in the *rdd* mutant resulted in downregulation of many stress response genes enriched for transposon or repeat sequences in their promoter regions. Methylation analyses indicate that these transposon and repeat sequences are the target of DNA demethylases and can play a significant role in the regulation of defence-related genes.

## Results

### The *rdd* mutant shows enhanced susceptibility to *Fusarium oxysporum*

**Figure 1:**
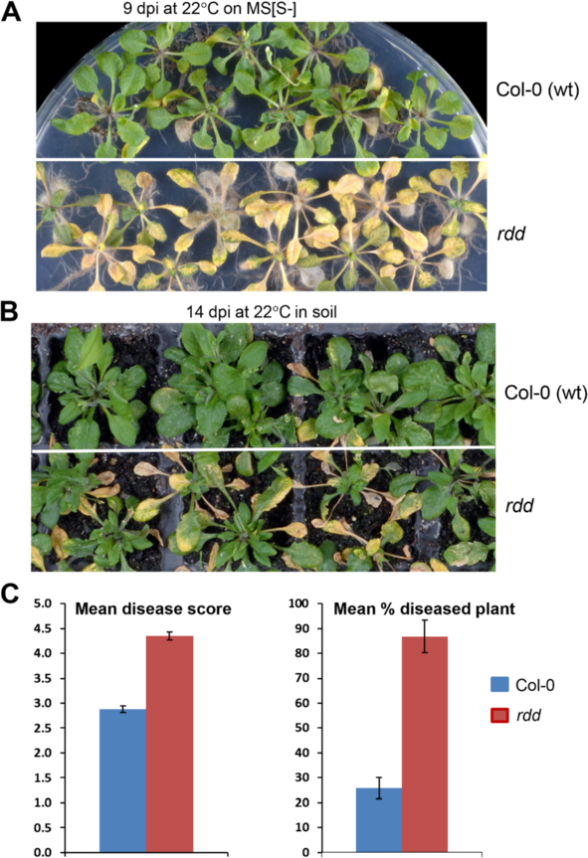
***rdd*** **plants are more susceptible to** ***F. oxysporum*** **than WT Col-0 plants. (A)** Col-0 and *rdd* plants infected and grown on sucrose-free MS plate. **(B)** Col-0 and *rdd* plants infected and grown in soil. **(C)** Disease symptom scores based on either the number of leaves showing chlorosis (left; 0 = non-infection, 1 = 1 to 3 leaves showing chlorosis, 2 = 4 to 6 leaves showing chlorosis; 3 = 7 to 9 leaves showing chlorosis; 4 = all leaves showing chlorosis; 5 = dead plant) or the percentage of plants showing a disease score of 4 or 5 (right).

Three-week-old wild-type (WT) Col-0 plants and *rdd* mutant plants were inoculated with *F. oxysporum* f. sp. *conglutinans* (*Fo*) by root dipping and grown on either sucrose-free MS agar (MS[S-]) or in soil. The *rdd* plants showed enhanced disease symptoms on MS[S-] with strong leaf chlorosis and fungal growth at 9 days post inoculation (dpi), whereas the Col-0 plants remained relatively healthy at the same time point (Figure 1A). Inoculation assays conducted on soil-grown plants showed similar results (Figure 1B), although symptom development was slightly delayed compared to plate inoculation assays. To quantify the severity of the disease development, we infected a large number of *rdd* and Col-0 plants on MS[S-] at 26°C, and scored the disease phenotypes based on either a disease rating scale or the number of severely diseased plants. At 10 dpi, Col-0 plants exhibited an average disease rating of 2.8 compared to 4.3 for *rdd* plants (Figure 1C, left). Similarly, only 25% of the Col-0 plants were found severely diseased compared to more than 85% of the *rdd* plants (Figure 1C, right). These results indicated that plant disease resistance is compromised in *rdd*.

As *F. oxysporum* is a soil-borne fungal pathogen, we investigated if the increased susceptibility to this pathogen is determined by the root or the shoot in *rdd*. We performed reciprocal graftings between Col-0 and *rdd* and inoculated the grafted plants with *Fo*. As shown in Additional file 1: Figure S1, the control grafts *rdd*/*rdd* and Col-0/Col-0 showed disease phenotypes similar to the respective ungrafted *rdd* and Col-0 plants (Figure 1); at 14 dpi, the inoculated *rdd*/*rdd* plants showed intense and uniform chlorosis, whereas the Col-0/Col-0 plants showed only mild chlorosis. The reciprocally grafted plants (*rdd/*Col-0 and Col-0/*rdd)* showed an intermediate disease phenotype between Col-0 and *rdd*, with more chlorosis than the Col-0/Col-0 graft but much less than the *rdd*/*rdd* graft. This result suggested that both roots and aerial tissues account for the disease phenotypes observed in the *rdd* mutant. Quantification of fungal biomass showed only a slight increase in the root and shoot tissues of *rdd* (Additional file 1: Figure S2), suggesting that the increased disease susceptibility observed in *rdd* is not due to enhanced presence of *Fo* in root tissues but is likely caused by increased sensitivity of the plant to disease symptom development.

### Many plant stress response genes are downregulated in the *rdd* mutant

To examine the molecular basis of increased susceptibility to *Fo* in the *rdd* mutant, we investigated gene expression changes in *rdd* in comparison to Col-0 by microarray analysis of uninfected plants (microarray data accession: GSE60508). A total of 348 genes (representing 374 gene probes) were differentially expressed (≥2-fold change) between *rdd* and Col-0, including 42 transposable element genes, seven pseudogenes, and 299 protein-coding genes of known or unknown function (Table 1 and Additional file 2: Table S1). The majority of protein-coding genes (248 out of 299 or 83%) were downregulated in the *rdd* mutant in comparison to Col-0. Real-time RT-PCR (RT-qPCR) analysis of 13 randomly selected genes verified the validity of the microarray data (Additional file 2: Table S2).

**Table 1:** **A large proportion of** ***rdd*** **-downregulated genes are stress response-related**

	**Upregulated in** ***rdd***	**Downregulated in** ***rdd***
Total differentially expressed accessions (fold change ≥2; *P* value <0.01 (n)	69 (average fold change = 2.5)	279 (average fold change = 3.5)
Transposable element gene	15	27
Pseudogene	3	4
Unknown protein	9	35
Genes of known function	42	213
Stress response genes identified by MapMan	12	99
All stress response genes identified	25	160

Gene Ontology (GO) term analysis was used to determine the functional classes of the 348 differentially expressed genes in *rdd*. The significantly enriched GO terms included extracellular region, endomembrane system, and flower development (Additional file 2: Table S3). Analysis of these 348 genes using MapMan [14] identified 111 genes that have known or putative functions in the biotic stress pathways (Table 1 and Additional file 2: Table S4). This represents approximately one-third of all differentially expressed genes. A further analysis using the MetGenMAP program [15] and TAIR 10 gene annotation information identified an additional 74 genes that have a potential function in stress responses including abiotic stress (Additional file 2: Table S5). Therefore, the total number of genes with known or putative stress response function amounted to 185 (Table 1), representing approximately 60% of all the differentially expressed genes (excluding TE genes). The majority (86%) of these known and potential stress response genes were downregulated in the *rdd* mutant (Table 1 and Additional file 1: Figure S3). Taken together, the microarray result suggested that DNA demethylases are required for the expression of these stress response genes in Arabidopsis, and that the reduced expression of these genes in *rdd* may contribute to the enhanced susceptibility to *Fo* infection.

### *rdd*-downregulated genes are enriched for *Fo* responsive expression patterns

**Figure 2:**
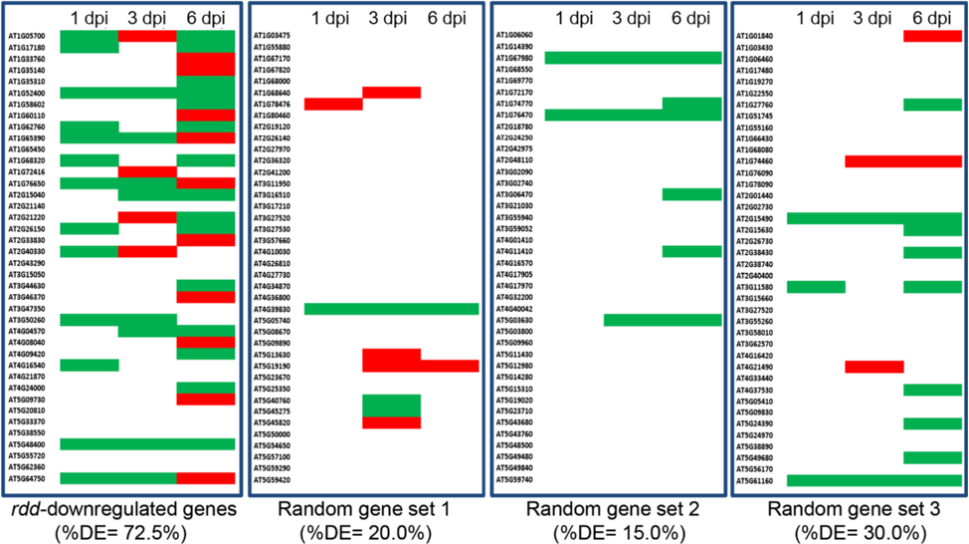
**The** ***rdd*** **-downregulated stress response genes are enriched for** ***Fo*** **-induced expression pattern.** Forty of the 99 *rdd*-downregulated genes identified by MapMan (Additional file 2: Table S4) have sequence reads in the RNA-seq data of *Fo*-infected and uninfected Col-0 plants at all three time points (1, 3, 6 dpi). Heatmap analysis revealed that 29 genes (72.5%) show ≥2-fold differential expression (DE) in *Fo*-infected plants in comparison to uninfected plants at one or more of the three time points (left). Three sets of 40 randomly selected genes (all having sequence reads in the RNA-seq data) show no enrichment for *Fo*-induced differential expression. Green bars, upregulation by *Fo* infection in the RNA-seq data; red bars, downregulation in the RNA-seq data.

We have previously investigated the transcriptome profiles of *Fo*-infected and uninfected Col-0 plants at 1, 3, and 6 dpi using RNA sequencing (RNA-seq) [16,17]. To examine the expression pattern of *rdd*-downregulated genes upon *Fo* infection, we first compared the 99 downregulated biotic stress response genes identified by MapMan (Table 1 and Additional file 2: Table S4) with the *Fo*-responsive gene sets identified in the RNA-seq analysis. Forty of these genes had reads in the RNA-seq datasets of all three time points. Heatmap analysis showed that the majority (72.5%) of these 40 genes were up- or downregulated by ≥2-fold at one or more of the three time points upon *Fo* infection (Figure 2 and Table 2). This is in contrast to three sets of 40 randomly selected genes (all showing RNA-seq reads at all three time points), of which less than 30% showed ≥2-fold differential expression upon *Fo* infection. Overlap of the expanded list of *rdd*-downregulated stress response genes (Additional file 2: Table S5) with the RNA-seq data showed a similar enrichment for *Fo*-induced up- or downregulation: 57 (71.2%) of the 80 genes that had RNA-seq reads showed ≥2-fold differential expression upon *Fo* infection, in contrast to 25% to 46% of the randomly selected genes (Additional file 1: Figure S4A and Table 2).

**Table 2:** ***Fo*** **-responsive expression pattern of** ***rdd*** **-downregulated genes in WT Col-0**

	**All** ***rdd*** **-downregulated genes**	***rdd*** **-downregulated stress response genes identified by MapMan**	**All** ***rdd*** **-downregulated stress response genes**
Total genes used in the analysis (n)	279	99	160
Genes showing RNA-seq reads at all three time points (n)	124	40	80
Genes showing ≥2-fold upregulation by *Fo* infection (n)	46	15	30
Genes showing ≥2-fold downregulation by *Fo* infection (n)	26	8	17
Genes showing mixed up- and downregulation by *Fo* infection (n)	14	6	10
Total genes showing ≥2-fold differential expression in *Fo*-infected plants (n)	86 (69.4%)^a^	29 (72.5%)	57 (71.2%)

^a^This is the percentage against the number of genes showing RNA-seq reads at all three time points.

We also tested the *Fo*-mediated alterations of all *rdd*-downregulated genes using the RNA-seq data. Of 279 genes downregulated in *rdd*, 124, which included eight TE genes, two pseudogenes, and 114 protein-coding genes, showed RNA-seq reads at all three time points. These 124 genes also showed enrichment for *Fo*-responsive genes, with 86 (69.4%) showing ≥2-fold up- or downregulation in the RNA-seq data in contrast to <31% for three randomly selected gene sets (Additional file 1: Figure S4B and Table 2). This result indicated that, in addition to the known and putative stress response genes listed in Additional file 2: Table S5, a substantial number of the remaining genes downregulated in *rdd* are responsive to *Fo* infection, suggesting that they may also have a role in stress response. It is interesting to note that the proportion of the *rdd*-downregulated genes that were induced in *Fo*-infected Col-0 plants was much higher than the number of genes repressed upon *Fo* infection (Table 2).

The same heatmap analysis was also performed to determine if genes upregulated in *rdd* were enriched for *Fo*-responsive genes. Only 21 of the 70 upregulated genes had reads in the RNA-seq data at all three time points, of which 10 (47.6%) showed differential expression (data not shown). This suggested that the *rdd*-upregulated genes are not highly enriched for *Fo*-responsive genes, although the number of genes is too small to draw a significant conclusion. Taken together, the heatmap analysis showed that genes downregulated in *rdd* are enriched for *Fo*-responsive expression in Arabidopsis, suggesting that they play a role in *Fo* resistance.

### *Fo*-responsive expression is compromised in *rdd* in comparison to Col-0

**Figure 3:**
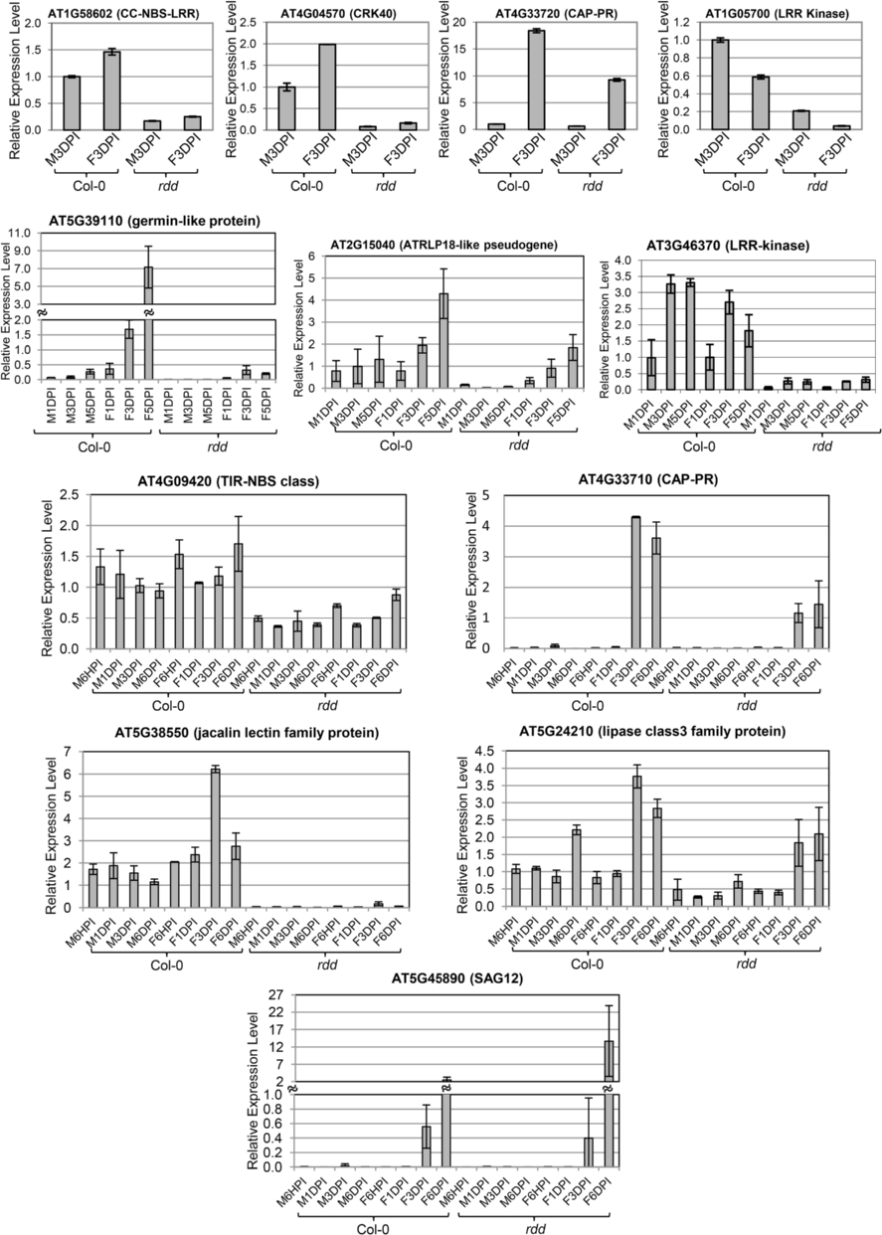
**RT-qPCR analysis of** ***rdd*** **-downregulated stress response genes.** The *Actin 2* gene was used as the internal reference. DPI, days post inoculation; F, *Fo*-inoculated; M, mock-inoculated.

We investigated the expression pattern of 11 *rdd*-downregulated genes with known or putative stress response function (Table 3) in *Fo*-infected Col-0 and *rdd* plants using RT-qPCR. Consistent with the microarray data, all 11 genes were downregulated in the mock-treated *rdd* mutant compared to Col-0 plants (although for *SAG12* this is not obvious in Figure 3 because of its low level of expression in uninfected plants) (Figure 3 and Additional file 1: Figure S5). The expression patterns in *Fo*-infected Col-0 can be classified into three types: (1) upregulated by *Fo* infection, which represented the majority (eight genes); (2) downregulated by *Fo* infection (the two LRR kinase genes); and (3) largely unaffected by *Fo* infection (the TIR-NBS gene) (Table 3, Figure 3, and Additional file 1: Figure S5). In *rdd* plants, these *Fo*-responsive expression patterns were largely retained for these genes (Figure 3 and Additional file 1: Figure S5). Thus, if the genes were induced in Col-0 by *Fo*, they were also induced in the *rdd* plants (for example, *CAP-PR* and *CRK40*). Similarly, if the genes were downregulated in Col-0 by *Fo*, they were also downregulated in *rdd* (for example, *LRR Kinase*, AT1G05700). However, the level of gene expression remained lower in *rdd* than in Col-0 upon *Fo* infection except for *SAG12*, which appeared to be more upregulated in *rdd* at 6 dpi. In addition to these 11 genes, we analyzed the expression pattern of AT4G33710 (another *CAP-PR*) located less than 1 kb upstream of AT4G33720 (*CAP-PR*) in the same orientation, which did not show differential expression in the microarray experiment possibly due to low-level signals in both *rdd* and Col-0. This gene, like AT4G33720, was also more induced by *Fo* in Col-0 than in *rdd* (Figure 3). Thus, in *rdd* these stress response genes in general did not achieve the level of *Fo*-responsive expression seen in WT Col-0 plants, suggesting that DNA demethylases are involved in the stress-related expression of these genes.

**Table 3:** ***rdd*** **-downregulated stress response genes analyzed by RT-qPCR**

**Accessions in TAIR**	**Annotation**	**Log2 fold change in** ***rdd*** **microarray**	**Response to** ***Fo*** **infection in Col-0**
AT1G05700	Leucine-rich repeat kinase (LRR kinase)	-1.07	Down
AT1G58602	LRR and NB-ARC domains-containing disease resistance protein (CC-NBR-LRR)	-3.31	Slightly up
AT2G15040	Pseudogene for receptor-like protein (ATRLP18)	-2.54	Slightly up
AT3G46370	Leucine-rich repeat kinase (LRR kinase)	-3.18	Down
AT4G04570	Cysteine-rich receptor-like kinase (CRK40)	-3.28	Up
AT4G09420	Disease resistance protein (TIR-NBS class)	-1.13	No clear change
AT4G33720	Pathogenesis-related protein of CAP superfamily (CAP-PR)	-3.54	Up
AT5G38550	Jacalin lectin family protein	-4.36	Slightly up
AT5G39110	Germin-like protein	-2.44	Up
AT5G24210	Lipase class 3 family protein	-3.22	Slightly up
AT5G45890	Senescence-associated gene 12 (SAG12)	-3.77	Up
AT4G33710^a^	Pathogenesis-related protein of CAP superfamily (CAP-PR)	No differential expression due to background level expression	Up

^a^This gene is included because it is located upstream of AT4G3320 in the same orientation.

### Genes downregulated in *rdd* are enriched for transposable element sequences in their promoters

A significant number of the *rdd*-downregulated genes are TE genes (Table 1 and Additional file 2: Table S1), suggesting that TEs are major targets of DNA demethylases, consistent with TEs being usually repressed by DNA methylation. We surveyed the genomic regions of the *rdd*-downregulated stress response genes identified in the MapMan analysis for the presence of TE in the natural transposon and transposable element tracks of the TAIR10 genome browser. These regions included promoters (either defined as promoters in the starPRO DB database [18] or 2 kb upstream of the annotated transcription start site in TAIR10 if the promoter was not defined), the gene body (transcribed region), and 2 kb downstream of the gene body. These genes (Table 4 and Additional file 2: Table S4), particularly the most downregulated genes, are enriched for TE sequences in their promoters. For instance, the eight top-ranking genes (that is, with the highest fold values of downregulation in *rdd*) all have one or multiple TE sequences in their promoters, and 16 of the 24 genes (66.7%) with ≥4-fold downregulation in *rdd* have promoter TE sequences (Table 4 and Additional file 2: Table S4). In contrast, only about 30% of two sets of randomly selected genes had promoter TEs (Table 4). There is no apparent enrichment for TE sequences in the gene body or downstream regions of the genes (Table 4). This result suggested that promoter TE sequences may be important for the regulation of the *rdd*-downregulated stress response genes. These promoter TEs sequences are relatively short, mostly less than 1 kb in size, with an average size of around 500 bp. There is no clear difference between the *rdd*-downregulated genes and the randomly selected genes in the type of TE present either in the promoter or across the whole gene region, with the RC/Helitron type being the most highly represented, followed by the DNA/MuDR, LTR/Gypsy, LTR/Copia, DNA/HAT, and LINE/L1 classes (Additional file 1: Figure S6).

**Table 4:** ***rdd*** **-downregulated stress response genes are enriched for promoter TE sequences**

	**Total genes surveyed (n)**	**Genes with promoter TE (n)**	**Genes with 3′ TE (n)**	**Genes with gene body TE (n)**
All Stress response genes from MapMan analysis	99	45 (45.4%)	15 (15.2%)	3 (3.0%)
≥4-fold downregulated stress response genes	24	16 (66.7%)	1 (4.2%)	1 (4.2%)
2-4-fold downregulated stress response genes	75	29 (38.7%)	14 (18.7%)	2 (2.7%)
Random gene set 1	77	23 (29.9%)	10 (13.0%)	1 (1.3%)
Random gene set 2	130	42 (32.3%)	11 (8.5%)	2 (1.5%)

### Downregulation of stress response gene expression in *rdd* is associated with DNA methylation changes around promoter TE sequences

**Figure 4:**
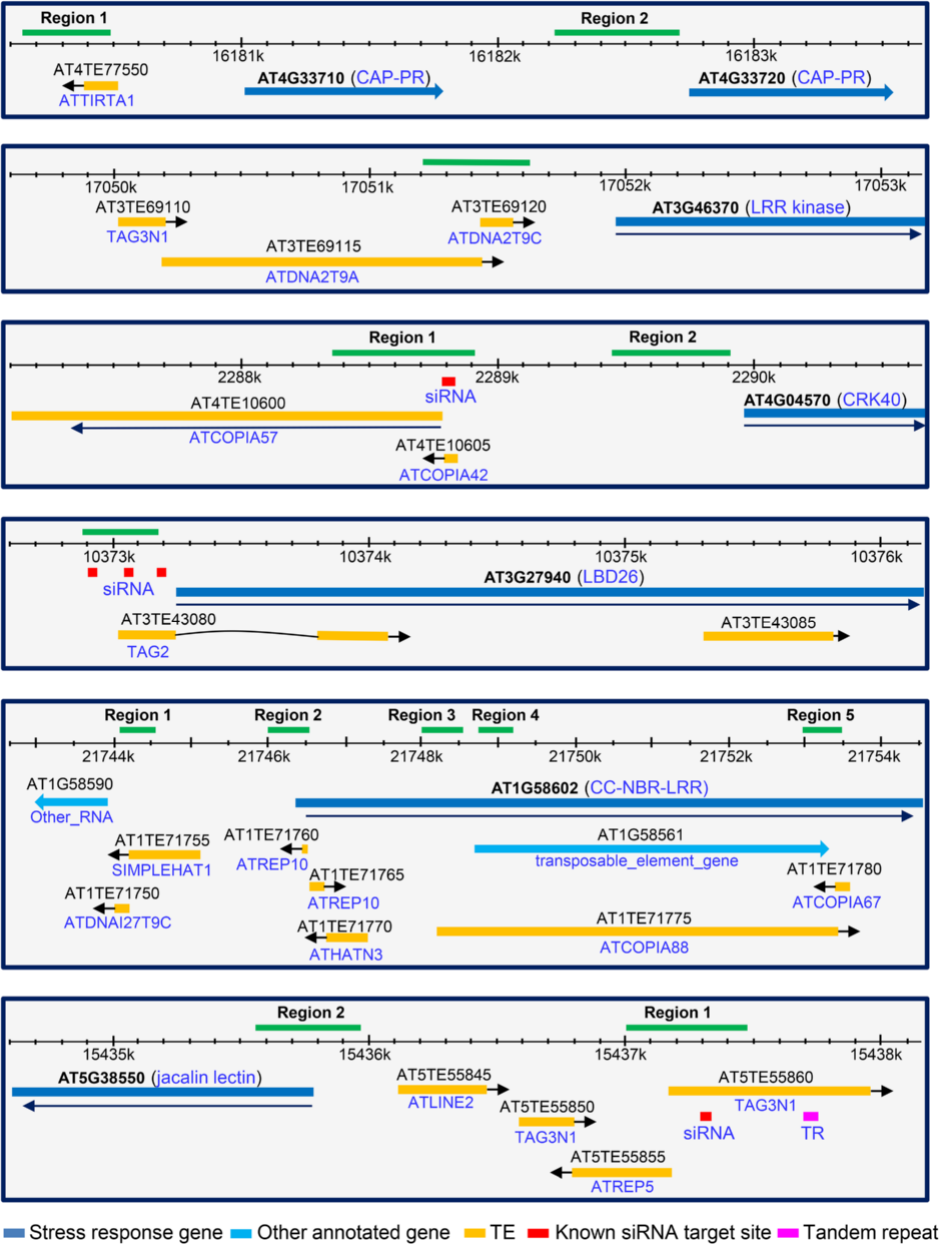
**Promoter features of genes analyzed by bisulfite sequencing.** The green lines indicate the bisulfite-sequenced regions. The coordinates for the genomic location of these sequenced regions are given in Additional file 2: Table S10 together with bisulfite PCR primer sequences.

To investigate if promoter TE sequences in the stress response genes are targeted by DNA demethylases, we performed bisulfite sequencing analysis to examine the methylation status of six *rdd*-downregulated stress response genes (Figure 4) in *Fo*-infected and uninfected Col-0 and *rdd* plants at 1, 3, and 6 dpi. All 24 DNA samples (two biological replicates each of the 1, 3, and 6 dpi plant samples used for the RT-qPCR analysis in Figure 3, bottom 5 panels) were efficiently converted by bisulfite, as indicated by a complete lack of cytosines in the bisulfite PCR product of the Arabidopsis chloroplast *psaA* gene (for example, Additional file 1: Figure S7A), known to have no DNA methylation [19]. To determine the DNA methylation level in the genes of interest (Figure 4), we sequenced the PCR product from each bisulfite-treated sample as a mixed DNA population, and measured cytosine methylation levels by calculating the ratio between the peak values of cytosine (C) and thymine (T) residues in the sequencing trace files. This approach was expected to be preferable to sequencing individual *E. coli* plasmid clones, as the latter can be affected by uneven cloning efficiency of different DNA sequence variants in a bisulfite PCR product population. To confirm the validity of this approach, we sequenced 40 individual pGEM-T Easy clones of PCR product from Region 2 of gene AT1G58602, and compared the result with that of directly sequencing the PCR product population. The two approaches gave a comparable pattern of methylation changes across the sequence (Additional file 1: Figure S8).

#### TEs are the target for DNA methylation

**Figure 5:**
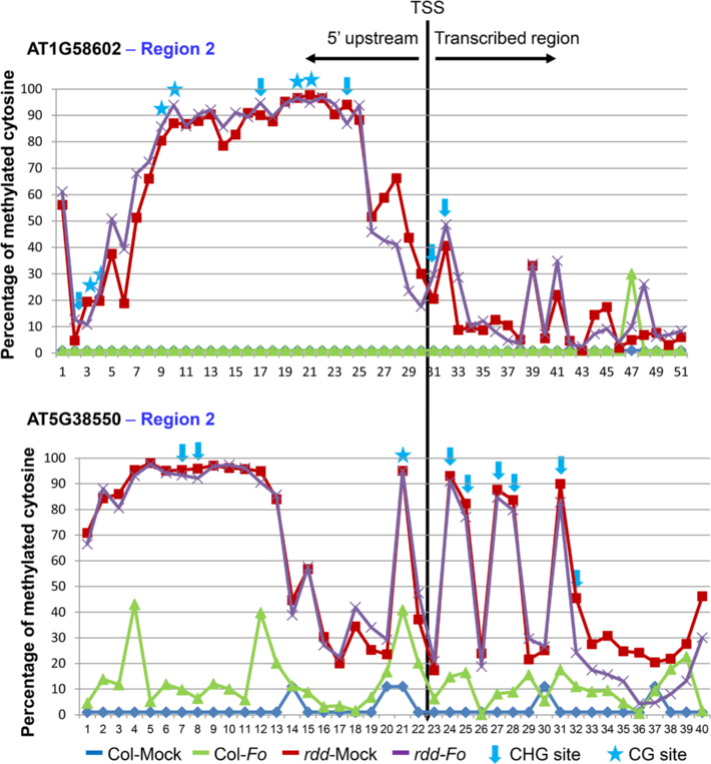
**Methylation pattern of the region near transcription start site (TSS) in AT1G58602 (CC-NBS-LRR) and AT5G38550 (jacalin lectin).** The black vertical line indicates the junction between promoter and TSS. CG and CHG sites are indicated by asterisks and downward arrow, respectively. The remaining points on the line indicate CHH sites. *Fo*, *F. oxysporum-*infected.

**Figure 6:**
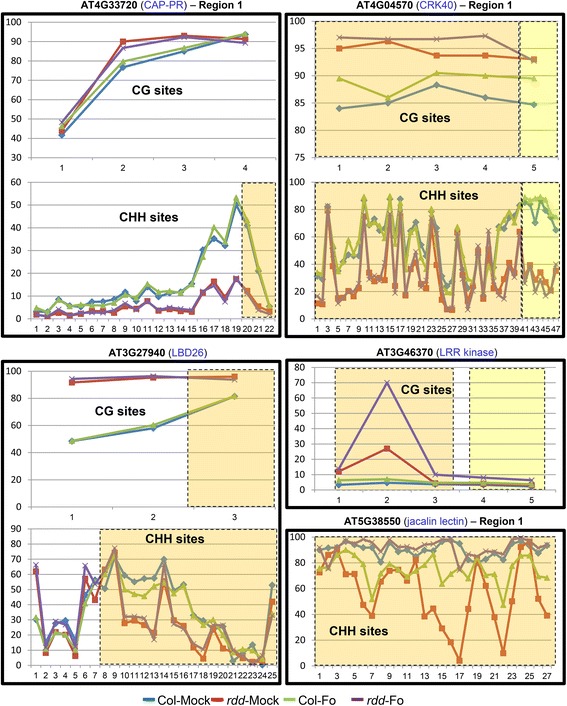
**CG and CHH methylation patterns in Col and** ***rdd*** **plants.** The X-axis indicates the individual cytosines in CG or CHH contexts in a sequential 5′ to 3′ order along the bisulfite-sequenced region, and the Y-axis shows the percentage of methylated cytosines at each CG or CHH position. The color-shaded areas indicate the TE regions, with yellow and orange colors representing two different TEs.

Regions that overlap with, or are within 200 bp of, TE sequences either in the promoter or gene body (Figure 4) showed cytosine methylation in *rdd* and/or Col-0 in all six genes (Figures 5 and 6). In contrast, the two regions that are distal to TE sequences, namely Region 2 in AT4G33720 (*CAP-PR*) (approximately 1.6 kb from TE) and AT4G04570 (*CRK40*) (approximately 500 bp from TE), had no cytosine methylation in either *rdd* or Col-0 samples (Additional file 1: Figure S7B). This indicated that TEs and their surrounding sequences in these *rdd*-downregulated stress response genes are specifically targeted for DNA methylation.

#### Promoter TE and surrounding sequences show methylation changes in *rdd*

The bisulfite sequencing revealed methylation changes between *rdd* and Col-0 in promoter TEs and the surrounding sequences (Figures 5 and 6), suggesting that these TEs are targeted by DNA demethylases. Most of these changes, particularly in CHH methylation, occurred in a localized manner, spanning a short segment of the sequenced regions. Overall, three patterns of methylation changes were observed between *rdd* and Col-0. First, little or no methylation was detected in Col-0 but strong methylation occurred in all sequence contexts in *rdd* (Figure 5). The two sequences that showed this pattern of methylation changes, Region 2 of AT1G58602 and AT5G38550, are both located immediately upstream of the transcription start site (TSS). Thus, for these two genes, the reduced expression in *rdd* can be directly associated with a gain of DNA methylation in their proximal promoter sequences. This result suggests that DNA demethylases are required to prevent promoter methylation and maintain active expression of these genes in WT plants. Interestingly, the TE sequence overlapping with Region 2 of AT1G58602 is inside the 5′ UTR, but strong methylation in *rdd* only occurred upstream of TSS with a clear demarcation at TSS separating the methylated promoter sequence and unmethylated 5′ UTR sequence (Figure 5). Furthermore, while both the upstream and 5′ UTR areas of AT5G38550 Region 2 showed strong methylation in *rdd*, only the upstream area had strong methylation in all sequence contexts, including CHH methylation (Figure 5). These results suggest that DNA demethylases preferentially affect the methylation of promoter sequences. Consistent with this view, the three bisulfite sequenced regions that overlap with the long TE in the gene body of AT1G58602, namely Region 3, Region 4, and Region 5 (Figure 4), showed no significant differences in methylation between *rdd* and Col-0 (Additional file 1: Figure S9).

Two other patterns of methylation changes around promoter TEs in *rdd* include: (1) genes exhibiting an increase in CG methylation levels with a concurrent decrease in CHH methylation levels; or (2) genes exhibiting either an increase in CG methylation or a decrease in CHH methylation only (Figure 6). In the former group, three *rdd*-downregulated genes, AT4G33720 (*CAP-PR*), AT4G04570 (*CRK40*), and AT3G27940 (*LBD26*), showed both increased CG methylation and reduced CHH methylation in *rdd* in comparison to Col-0 (Figure 6). In the latter group, Region 1 of AT5G38550 (jacalin lectin) showed a clear reduction in CHH methylation in mock-treated *rdd* (Figure 6), but the CG sites were almost fully methylated in both *rdd* and Col-0 (Additional file 1: Figure S10A). Similarly, AT3G46370 (*LRR kinase*) had almost no CHH methylation in any of the samples (Additional file 1: Figure S10A) but showed an increase in CG methylation (Figure 6). Thus, for the latter two genes, their downregulation in *rdd* was correlated with either increased CG methylation or decreased CHH methylation. In contrast to CG and CHH methylation changes, no clear difference in CHG methylation was observed between *rdd* and Col-0 (Additional file 1: Figure S10B).

It is interesting to note that sequences that are either overlap with or are adjacent to the promoter TEs showed stronger changes in CHH methylation than those further away from the TEs (for example, AT4G33720 and AT3G27940; Figure 6). This close association of CHH methylation changes with TEs further suggests that these promoter TEs are specifically targeted by DNA demethylases.

#### Fo *infection causes methylation changes but only in specific genes*

Methylation levels were in general not clearly affected by *Fo* infection in either *rdd* or Col-0. However, methylation at the CG site in AT3G46370 (LRR kinase) was increased upon *Fo* infection in *rdd* (Figure 6). For AT5G38550 (jacalin lectin), CHH methylation was reduced in Col-0 by *Fo* infection, but strongly increased in *rdd* by *Fo* infection (Figure 6 and Additional file 1: Figure S7C). The remaining genes analyzed did not show clear methylation differences between *Fo*-infected and uninfected plants, although subtle methylation changes were observed at some individual CHH sites for AT4G33720 and AT4G04570, with *Fo*-infected Col-0 showing slightly more CHH methylation than mock-treated plants (Figure 6). Taken together, these results suggest that *Fo* infection can induce dynamic methylation changes around promoter TE sequences, but these changes occur in a gene-specific manner.

### Many *rdd*-downregulated stress response genes are also repressed in RdDM mutants

**Figure 7:**
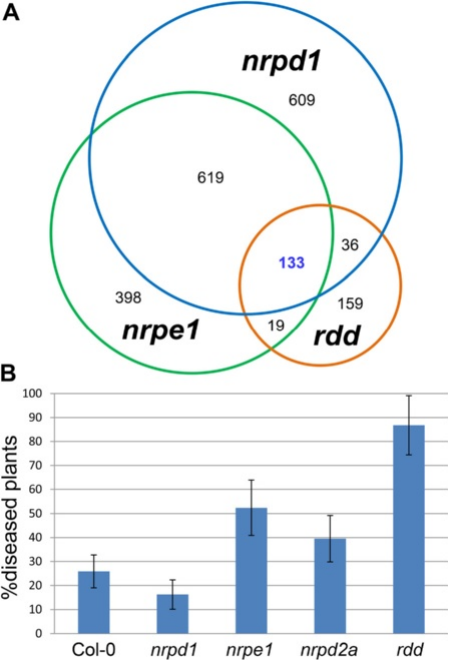
**Potential interaction between DNA demethylases and RdDM in Arabidopsis response to** ***Fo*** **. (A)** Overlap of differentially expressed genes between *rdd* and the RdDM mutants. The number in blue (133) represents genes differentially expressed in both the *rdd* mutant and the two RdDM mutants. Note that the total numbers of differentially expressed genes for the three mutants (1,397, 1,169, and 347 for *nrpd1*, *nrpe1*, and *rdd*, respectively) are slightly smaller than those listed in Additional file 2: Tables S1, S6, and S7 (1,399, 1,170, and 348), missing a few accessions representing variant gene models. This is because the VirtualPlant program [20] does not include all Arabidopsis gene models. **(B)** The Pol V mutant *nrpe1* shows increased susceptibility to *Fo* infection whereas the Pol IV mutant *nrpd1* is slightly more resistant to *Fo* than Col-0. The *nrpd2a* mutant, where both Pol IV and Pol V functions are disrupted, shows an intermediate disease response phenotype between *nrpd1* and *nrpe1*. A total number of 84 to 174 plants were assayed for each line.

The correlation between the reduced CHH methylation and the downregulation of some of the stress response genes in *rdd* suggests that CHH, or RdDM, may interact with DNA demethylases to regulate stress response gene expression. To investigate this possibility, we compared the microarray gene expression data of *rdd* with those of the RdDM mutant *nrpd1* (a Pol IV mutant) and *nrpe1* (a Pol V mutant) (microarray data accession: GSE60508). The number of differentially expressed genes in each mutant (relative to Col-0) were: *rdd* (348 genes; Additional file 2: Table S1), *nrpd1* (1399; Additional file 2: Table S6), and *nrpe1* (1170; Additional file 2: Table S7). A much larger set of genes was differentially expressed in the two RdDM mutants than in *rdd*, suggesting that the RdDM pathway targets a broader range of genes than DNA demethylases in Arabidopsis. Comparison of the three gene lists was performed using the Sungear software on VirtualPlant [20] to identify commonly regulated genes. As expected, a large number of differentially expressed genes (619 + 133 = 752) were shared between *nrpd1* and *nrpe1* (Figure 7A). Among all three mutants, 133 genes were commonly differentially expressed (Figure 7A and Additional file 2: Table S8). This number represents 38% of all the differentially expressed genes in *rdd*, indicating a strong overlap between *rdd*-regulated genes and RdDM-regulated genes. Of the 133 genes, 70 are stress response genes listed in Additional file 2: Table S5 (highlighted in red in Additional file 2: Table S8). Importantly, over 91% of the 133 common genes were downregulated in all three mutants, a proportion that is much higher than the total downregulated genes in the individual mutants, namely 80.2% in *rdd*, and 60.2% in both *nrpd1* and *nrpe1* (Table 5). These results suggest that DNA demethylases interact with RdDM to maintain or positively regulate stress response gene expression in WT plants. Consistent with this, the RdDM pathway mutants *nrpe1* (a Pol V mutant) and *ocp11* (an AGO4 mutant) both showed increased susceptibility to *Fo* infection in comparison to Col-0 (Figure 7B and Additional file 1: Figure S11). The disease symptoms in *nrpe1*and *ocp11* were less severe than in *rdd* (Additional file 1: Figure S11). The *nrpd2a* mutant, in which both Pol IV and Pol V function is disrupted, also showed a slightly increased disease phenotype compared to Col-0 (Figure 7B). However, the Pol IV mutant *nprd1* showed no increase in disease susceptibility, and was slightly more resistant to *Fo* than Col-0 (Figure 7B). This result is consistent with a previous report showing that RdDM mutants of the downstream Pol V complex, including *nrpe1*, *ago4*, *drd1*, and *drm1drm2*, but not the upstream Pol IV mutant *nrpd1*, showed increased disease susceptibility to the necrotrophic fungal pathogens *Botrytis cinerea* and *Plectosphaerella cucumerina* [10]. This suggests that DNA demethylases may interact with RdDM via the downstream Pol V complex. The relatively mild *Fo*-mediated symptoms of *ago4*, *nrpe1*, and *nrpd2a* in comparison to *rdd* could be due to the RdDM pathway targeting a broader range of genes than the demethylases that primarily target stress response genes. Furthermore, the expression of over half of the *rdd*-affected stress response genes listed in Additional file 2: Table S5, including most of the genes analyzed in Table 3 and Figure 4, was unaffected in the *nrpd1* and/or *nrpe1* mutants, which could also in part account for the reduced symptom severities of the RdDM mutants in comparison to *rdd*.

**Table 5:** **The large majority of commonly differentially expressed genes are downregulated in both** ***rdd*** **and the two RdDM mutants**

	**Total differentially expressed genes (≥2-fold) (n)**		**Commonly differentially expressed genes (n)**	
	Up	Down	Up	Down
*rdd*	69	279 (80.2%)^a^	7	126 (94.7%)^b^
*nrpd1*	556	843 (60.2%)	12	121 (91.0%)
*nrpe1*	465	705 (60.2%)	11	122 (91.7%)

^a^Percentage against total differentially expressed genes in each of the mutants.

^b^Percentage against the 133 commonly differentially expressed genes.

## Discussion

Previous studies have implicated DNA demethylation in plant biotic and abiotic stress responses [3]. For instance, *P. syringae* infection results in genome-wide reduction of DNA methylation in Arabidopsis [11]. In tobacco, treatment with aluminum, NaCl, cold or oxidative stress results in rapid reduction in DNA methylation in the coding region of a glycerophosphodiesterase-like gene [21]. Global demethylation of the rice genome with 5-azadeoxycytidine enhances bacterial resistance to virulent strains of *Xanthomonas* [22]. Similarly, a number of mutants deficient in DNA methylation show increased resistance to biotic stress including *P. syringae* infection [8]. A recent study provided a more direct evidence for the involvement of DNA demethylation in biotic stress response, showing that the *ros1* mutant had a higher level of accumulation and vascular spread of *P. syringae* than WT Arabidopsis [12]. This study also showed that treatment of Arabidopsis with flg22, a conserved peptide from the bacterial flagellum resulted in progressive demethylation and increased transcription of several retrotransposon sequences during the first few hours of treatment. Furthermore, a flg22-inducible disease resistance gene is downregulated in *ros1*, and this downregulation is associated with an increase in DNA methylation at a TE sequence near the transcription start site, implicating TE sequences in DNA demethylation-mediated gene regulation.

### DNA demethylases play a role in *Fusarium oxysporum* resistance by regulating stress response genes

We have shown that the triple demethylase mutant *rdd* is highly susceptible to *Fo*-induced disease symptoms. This mutant is defective in ROS1, DML2, and DML3 that are thought to account for all demethylation activity in somatic tissues in Arabidopsis, and displays much stronger DNA hypermethylation than any of the three single demethylase mutants [6]. Indeed, *rdd* plants showed stronger response to *Fo* infection than the *ros1*, *dml2*, and *dml3* single mutant plants (data not shown).

Using microarray analyses, we identified 348 genes that show >2-fold differential expression between *rdd* and Col-0. Importantly, over half of these differentially expressed genes have known or putative functions in stress responses, and the majority of them are downregulated in *rdd.* This raises the possibility that these DNA demethylases function primarily in maintaining or positively regulating stress response gene expression. Consistent with this, the *rdd* mutant has no visible developmental defects under normal growth conditions. Furthermore, previous studies have shown that no widespread DNA methylation or gene expression changes occur in *rdd*, with only 179 genes showing hypermethylation [6] and 167 genes showing differential expression [7] in comparison to WT Arabidopsis. Interestingly, there is very little overlap between the differentially methylated and differentially expressed gene lists from these two studies, with only two genes in common (Additional file 1: Figure S12B). These two gene lists also show low-level overlap with the 348 genes identified in our study, having only 10 and six common genes, respectively (Additional file 1: Figure S12B). No gene was found to be common among all three gene lists. This low level of overlap is likely due to different plant materials (plants with no roots, immature floral buds, and whole plants including roots, respectively) and techniques (genome tiling array, mRNA-seq and expression RNA microarray, respectively) being used in these three studies (Additional file 1: Figure S12A). The 348 differentially expressed genes identified in our study represent a significant expansion of target gene lists for DNA demethylases in Arabidopsis. Notably, the 10 genes shared between our gene list and that of Penterman *et al.* [6] are enriched for stress response functions. Of these, seven have known or putative function in stress response, two encode unknown proteins, and one is a retrotransposon gene (Additional file 1: Figure S12B). All 10 genes showed hypermethylation in *rdd* [6], and all, except the retrotransposon gene, were down-regulated in *rdd* (Additional file 2: Table S1). This is consistent with a primary function of DNA demethylases in the control of stress response gene expression.

### Promoter TE sequences appear to be the main target of DNA demethylases

The *rdd*-downregulated stress response genes are enriched for transposable element (TE) sequences in the promoter region. Bisulfite sequencing of six *rdd*-downregulated genes showed that these TE and surrounding sequences, but not the sequences distal to TE, are methylated. This analysis also revealed that the promoter TE and surrounding sequences showed altered methylation in *rdd* mutants, suggesting that promoter TEs are specifically targeted by the DNA demethylases. This is consistent with previous studies showing that DNA demethylases preferentially target gene ends (namely the upstream and downstream flanking regions of genes) and short TE sequences around these gene flanking regions [4,6,7,23]. Studies in maize and Arabidopsis [24,25] have suggested that such near-gene TEs are important in the control of gene expression, and that highly methylated TEs tended to be associated with repressed gene expression. The study in maize [24] also showed that the closer a TE sequence is to the TSS, the stronger the effect it has on the expression of the gene. Our bisulfite sequencing analysis showed a general increase in CG methylation in *rdd* in comparison to Col-0 around promoter TEs. For AT1G58602 and AT5G38550, there was a strong gain of methylation in all sequence contexts in *rdd* around the TE near the TSS. In contrast, regions of AT1G58602 around the long TE inside the first intron and other TEs further upstream in the promoter sequence showed no methylation changes (Figure 4 and Additional file 1: Figure S9). These results support a role of near-gene TEs in demethylase-mediated gene regulation, and suggest that increased CG methylation or gain of methylation around promoter TEs may account for the repressed expression of at least some of these stress response genes in *rdd*.

### RdDM-associated CHH methylation appears to play a positive role in stress response gene expression

The bisulfite sequencing analysis of the six stress-response genes showed a generally reduced CHH methylation (hypo-^m^CHH) in the TE and TE-associated regions in the *rdd* mutant plants. To examine if additional *rdd*-downregulated stress response genes have hypo-^m^CHH, we surveyed the publically available genome-wide bisulfite sequencing data published by Stroud *et al*. [26], which was performed on 3-week-old leaf tissue (without roots) of a different *rdd* mutant [6]. Despite the slight variation in tissue type and mutant source, the genome-wide sequencing data also showed hypo-^m^CHH in the respective regions of the six genes except for the region in AT3G27940, which exhibits increased CHH methylation (Additional file 1: Figure S13A). Furthermore, additional 11 genes out of the 47 most downregulated stress response genes in *rdd* showed localized hypo-^m^CHH in their promoter regions, and almost all of these short hypo-^m^CHH regions correspond to 24-nt siRNA clusters and/or TEs (Additional file 2: Table S9; Additional file 1: Figure S13B). Reduced CHH methylation of short TE sequences has been previously observed in the endosperm of the DNA demethylase mutant *dme* [23,27]. It was suggested that this is due to the functional disruption of the Polycomb Repressive Complex 2 (PRC2), which requires DME activity to promote non-CG methylation in endosperm [23]. It has also been suggested that the hypo-^m^CHH in the endosperm of *dme* could be caused by repressed transcription of TEs resulting in reduced amounts of RNA template for RDR2 to synthesize dsRNA and hence 24-nt siRNAs [4,27].

Similar mechanisms could be responsible for the hypo-^m^CHH in *rdd*. Thus, the reduced CHH methylation around promoter TE sequences in *rdd* could be a consequence of repressed TE transcription or disrupted PRC2 function but may have no direct role in the expression of the stress response genes. However, a recent study on *de novo* cytosine methylation in maize suggests that near-gene CHH methylation may play a positive role in gene expression [28]. This study shows that CHH methylation is different to CG and CHG methylation, and is enriched near gene ends where CG and CHG methylation are depleted. Significantly, while CG and CHG methylation exhibits an inverse correlation with gene expression, CHH methylation flanking genes shows a positive correlation with gene expression level. Bisulfite sequencing showed that the extent of the CHH methylation decrease was much greater than that of CG methylation increase for the two genes AT4G33720 and AT4G04570 (Figure 6). For Region 1 of AT5G38550, no significant CG methylation change was detected in *rdd* (Additional file 1: Figure S10), in contrast to the strong reduction in CHH methylation (Figure 6). These results suggest a positive role of CHH methylation in the control of these genes.

CHH methylation around gene-rich areas in Arabidopsis is induced and maintained by 24-nt siRNA-dependent RdDM. Indeed, the 17 stress response genes that display hypo-^m^CHH in *rdd* in either this study or the bisulfite sequencing data by Stroud *et al*. [26] generally show hypo-^m^CHH in the RdDM mutants *nrpd1* and *nrpe1* in the same regions (Additional file 2: Table S9). Consistent with CHH methylation playing a positive role in stress response gene regulation, *nrpe1* and *ocp11*, lacking the function of the downstream RdDM factors Pol V and AGO4, respectively, displayed enhanced susceptibility to *Fo* infection. Furthermore, the RdDM mutants *nrpe1* and *nrpd1* shared a large number of differentially expressed genes with *rdd*, and most of these genes were downregulated in all three mutants. However, unlike *nrpe1* and *ago4*, the *nrpd1* mutant did not show increased disease susceptibility to *Fo*, and this is consistent with the previous study by López *et al*. [10] suggesting that the Pol V complex, but not Pol IV, of the RdDM pathway is required for plant immunity. Interestingly, a genome-wide analysis of Pol V-dependent 24-nt siRNAs suggests that Pol V preferentially targets short (approximately 238 bp) intergenic TE sequences located in dispersed genomic regions [29], a feature that resembles DNA demethylases. This is different to Pol IV, which is required for the biogenesis of 24-nt siRNAs from the majority of RdDM target loci and therefore has a broader range of targets in the Arabidopsis genome [1,2]. This raises the possibility that DNA demethylases may interact with the downstream Pol V complex, but not the upstream siRNA biogenesis components, of RdDM to positively regulate the expression of stress response genes that have short TE sequences in the promoters. It is unclear how RdDM or CHH methylation can positively regulate gene expression. One possibility is that CHH methylation, or 24-nt siRNAs triggering CHH methylation, is involved in the recruitment of DNA demethylases to their specific targets, and therefore acts via DNA demethylases to regulate gene expression. Alternatively, RdDM or CHH methylation may play a role in repressing cryptic transcription initiated from a TE promoter sequence that would otherwise interfere with normal transcription of the adjacent gene. A previous study has suggested that silencing of TEs near genes plays a role in preventing the production of aberrant transcripts via read-through transcription beyond TE termini [30]. However, direct evidence for either of the scenarios is lacking.

### Are DNA demethylases involved in *Fusarium oxysporum*-inducible expression of stress response genes?

Our results show that the *rdd*-downregulated stress response genes are highly enriched for *Fo*-responsive expression patterns, with more genes being upregulated than downregulated upon *Fo* infection. Notably, almost all of the *rdd*-downregulated stress response genes that show *Fo*-responsive expression at the early time point (1 dpi) in our previously reported RNA-seq data are upregulated by *Fo* infection at this time point (Figure 2 and Additional file 1: Figure S4A). This suggests that the DNA demethylases play an active role during the stress response process, particularly at the early time point, to positively regulate gene expression. Consistent with this, Yu *et al.* [12] showed that flg22 treatment of Arabidopsis triggers progressive demethylation in TE sequences during the first 9 h following the treatment. Dowen *et al.* [8] also showed that the Arabidopsis genome undergo dynamic DNA methylation changes including hypomethylation under various biotic stress conditions. Our bisulfite sequencing analysis detected only subtle changes in methylation around the promoter TE sequences upon *Fo* infection. For instance, AT5G38550 (jacalin lectin) showed reduced CHH methylation in Col-0 upon *Fo* infection (Figure 6). It also showed a slight reduction in CG and CHG methylation in Col-0 (Additional file 1: Figure S10). This reduction in methylation coincides with a slight upregulation of this gene upon *Fo* infection (Figure 3). AT3G27940 (*LBD26*) exhibited a slightly reduced methylation in some of the CHH and CHG sites but showed no change in CG methylation (Figure 6 and Additional file 1: Figure S10). For AT4G33720 (*CAP-PR*) and AT4G04570 (*CRK40*), there appeared to be a subtle increase in CHH methylation at individual CHH sites in *Fo*-infected Col-0. Interestingly, AT3G27940 (*LBD26*) was slightly downregulated in Col-0 by *Fo*-infection (data not shown), whereas AT4G33720 (*CAP-PR*) and AT4G04570 (*CRK40*) were both upregulated, which appears to be consistent with CHH methylation playing a positive role in gene expression. The remaining two genes (AT3G46370 and AT1G58602) showed no clear changes in DNA methylation in WT plants upon *Fo* infection. This could indicate that not all demethylase-targeted promoter TE sequences undergo methylation changes upon *Fo* infection, which would be consistent with the observation that different biotic stresses induce DNA methylation changes in different subsets of loci in Arabidopsis [8]. An alternative explanation for the low-level *Fo*-induced methylation changes in our analysis could be that the changes only occur in specific tissues such as roots or vasculature, which were not detected due to dilution of DNA from these tissues by the DNA from whole plant tissues used in this study. Genome-wide bisulfite sequencing of *Fo*-infected and uninfected Arabidopsis plants is needed to further understand the role of DNA demethylases in the *Fo*-inducible expression of the stress response genes. Also, a future study should examine tissue or cell-specific changes of DNA methylation in the promoter TE sequences upon *Fo* infection.

## Conclusions

**Figure 8:**
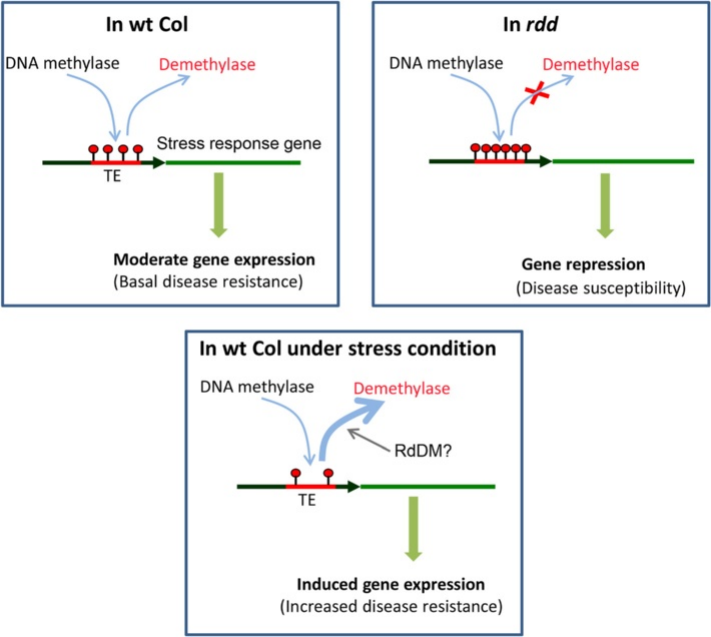
**A hypothetical model explaining how DNA demethylases regulate stress response genes by targeting promoter TE sequences and why** ***rdd*** **is susceptible to** ***Fo*** **infection.** In WT Arabidopsis (top left), both DNA methyltransferases (DNA methylase) and demethylases target TE sequences to maintain a steady-state level of methylation and a moderate level of gene expression. In *rdd* plants (top right), methylation levels are increased due to the absence of demethylases, resulting in repressed gene expression. Under stress conditions (bottom), DNA demethylase activity is increased (indicated by thicker arrow) due to unknown factors, resulting in reduced TE methylation and induced gene expression. RdDM interacts with DNA demethylases in this process but how this interaction occurs remains unknown.

Using *Arabidopsis thaliana* and *Fusarium oxysporum* as a plant-pathogen model, combined with gene expression and methylation analyses, we have generated evidence suggesting that DNA demethylases play an important role in fungal disease resistance by positively regulating the expression of a subset of stress response genes in Arabidopsis. Significantly, our results suggest that DNA demethylases target short promoter TE sequences to regulate these stress response genes. A question is why these stress response genes are regulated by such a TE-mediated mechanism. One possibility is that products of these genes, required for stress responses, are detrimental to plant development if accumulating at high levels, and therefore need to be repressed under normal growth conditions via promoter methylation. This is consistent with the existence of methylation in these promoter TE in WT plants, and with the generally low level of expression of these stress response genes in our microarray data. Under stress conditions, these genes are temporally activated through the action of DNA demethylases (to trim promoter TE methylation) to confer disease resistance or stress tolerance. This model (Figure 8), if proven, would make TEs an important genetic element in plant responses to environmental conditions. Our results also suggest a positive interaction between DNA demethylases and RdDM in the control of stress response genes, but how these pathways might interact remains to be further investigated.

## Materials and methods

### Plant growth and infection of Arabidopsis plants with *F. oxysporum*

Arabidopsis lines used in this study include WT Col-0, the triple *rdd* mutant (Col-0 background, derived from Salk_045303 Salk_056440 Salk_131712), the AGO4 mutant *ocp11*, the Pol IV mutant *nrpd1a-3* (*nrpd1*), and the Pol V mutant *drd3-7* (*nrpe1*). Seeds were sown on Murashige and Skoog (MS) [31] agar plates supplemented with 3% sucrose and incubated at 4°C for 3 days. Seedlings were transferred to fresh MS plates 1 week after germination and grown under 16 h light/8 h dark photoperiod at 22°C. Reciprocal grafts of Col-0 and *rdd* plants were performed as previously described [32]. Growth of *F. oxysporum* f. sp. *conglutinans* strain Fo5176 (obtained from Dr Roger Shivas, Queensland Department of Primary Industries and Fisheries, Australia) was performed as previously described [33]. Three-week-old plants were infected by dipping the roots in a 2 × 10^6^ spores/mL inoculum and replacing the plants on MS agar without sucrose or in soil as indicated. Plants were grown under 16 h light/8 h dark photoperiod at 22°C or 26°C. Plants grown at 26°C were scored for disease symptoms or for percentage of diseased plants (Figure 1) at 10 dpi. At least three independent experiments, using ≥20 plants per experiment, were performed.

### Microarray and gene ontology analyses

Total RNA from whole plant tissues of 3-week-old *A. thaliana* plants grown on MS agar was extracted using the RNeasy Midi Kit (Qiagen, Hilden, Germany). The number of biological replicates for each plant genotype were as follows: Col-0 (3 biological replicates), *rdd* (2), *nrpd1* (2), and *nrpe1* (2) respectively. RNA quality was analyzed using the Agilent 2100 Bioanalyzer (Agilent Technologies, Santa Clara, CA, USA) and samples with RNA Integrity Number (RIN) of ≥8.70 were used for the *A. thaliana* 12 × 135 K gene expression microarray (Nimblegen, Reykjavik, Iceland). The 12 × 135 K format allows simultaneously profiling of 12 samples on a single slide. Each array contains 60-mer probes targeting 39,042 genes (TAIR 9.0) with four probes per gene. Nimblegen analysis provides expression values normalized using the Robust Multichip Average algorithm [34] and these were further normalized between arrays using quantile normalization. Statistical testing between samples was performed using the LIMMA package [35] in Bioconductor and probes displaying ≥2-fold expression changes significant at the *P* ≤0.01 level were chosen for further analyses. Gene ontology analysis was performed in agriGO [36] using TAIR9 ontology annotations and the hypergeometric statistical analysis method with False Discovery Rate (FDR) correction for multiple testing. The background frequency of terms was calculated from the list of probe sets that showed signal 2 standard deviations greater than background frequency in at least one group of samples profiled. Terms were considered significantly enriched if they met statistical significance *P* <0.05 after correction for multiple testing. Differentially expressed genes with ≥2-fold change in gene expression were mapped onto diagrams of metabolic pathways or other biological processes by the MapMan software version 3.6.0RC1 [37] using Mappings and Pathways files from the TAIR10.0 database [38].

### Methylation analysis using bisulfite sequencing

Bisulfite conversion of Arabidopsis genomic DNA (approximately 2 μg) was performed as described in Wang *et al.* [39]. A total of 24 DNA samples, including two biological replicates each isolated from the same 1, 3, and 6 dpi plant samples used in the RT-qPCR analysis (Figure 3; the bottom 5 panels), were treated with bisulfite. The bisulfite-treated DNA was purified using Qiagen PCR Purification kit. Primer design (sequences shown in Additional file 2: Table S10), nested PCR and direct sequencing of PCR products were as described in Finn *et al.* [19]. Trace file data of the sequenced PCR products were opened using the BioEdit software [40], exported to Microsoft Excel using the ‘Export trace values (tab-delimited text)’ feature, and the relative peak heights of cytosines and thymines calculated to indicate the relative degree of methylation at each cytosine location.

### Real-time RT-PCR and statistical analyses

For gene expression analyses, 3-week-old plants were either mock-treated (water) or infected with *F. oxysporum* as above and placed on MS agar without sucrose for the indicated times. RNA from whole plant tissues was extracted as described above or using the phenol extraction method [41]. Synthesis of cDNA was performed using an oligo-dT primer and Superscript III reverse transcriptase (Invitrogen) according to the manufacturer’s instructions. RT-qPCR was performed in the Rotor-Gene 6000 (Corbett Life Science, San Francisco, CA, USA) real-time rotary analyzer using SYBR Green JumpStart Taq ReadyMix (Sigma, St. Louis, MO, USA) in three technical replicates for three independent biological samples. Primers used for RT-qPCR are listed in Additional file 2: Table S10. Transcript levels were measured using the comparative quantification method [42] and normalized against the house-keeping gene At3g18780 (*Actin 2*). For each infection time point, the transcript level in mock-treated Col-0 plants was used as the reference for normalization. Two-sample t-tests (assuming unequal variances) were performed using Excel (Microsoft, Washington, DC, USA) to determine significant (*P* value <0.05) differences in gene expression levels between treatments (mock versus infected) within the same genotypes or between different genotypes within the same treatment.

### Heatmap analysis

Gene expression data in reads per million were extracted from RNA-seq data generated by Zhu *et al.* [16]. Fold change (Mock/*Fo*) analysis were performed for all three time points (1, 3, and 6 dpi). In order to accurately illustrate changes in gene expression following *Fo* infection, genes with data missing (no reads detected) at one or more time points were excluded from heatmap analysis. Heatmaps were generated using Microsoft Excel. Random genes were selected using Selected random lines (version 2.0.1) in Galaxy [43].

## Additional files

Additional file 1
**Supplementary figures.**
Additional file 2
**Supplementary tables.**

## Abbreviations

AGO4: Argonaute 4
DML: Demeter-like
*Fo*: *F. oxysporum* f. sp. *conglutinans*
GO: Gene ontology analysis
Pol IV: RNA polymerase IV
Pol V: RNA polymerase V
*rdd*: *ros1 dml2 dml3* triple mutant
RdDM: RNA-directed DNA methylation
RNA-seq: RNA sequencing
ROS1: Repression of silencing
RT-qPCR: Real-time reverse transcriptase polymerase chain reaction
siRNA: Small interfering RNA
TE: Transposable element
TSS: Transcription start site
UTR: Untranslated region
WT: Wild type
