# Supplementary material for: DNA demethylases target promoter transposable elements to positively regulate stress responsive genes in Arabidopsis
Source: Genome Biol. 2014 Sep 17;15(9):458. doi: 10.1186/s13059-014-0458-3 (PMC4189188; doi:10.1186/s13059-014-0458-3)
Supplement: Additional file 1 — Supplementary figures. [file 13059_2014_458_MOESM1_ESM.pdf]

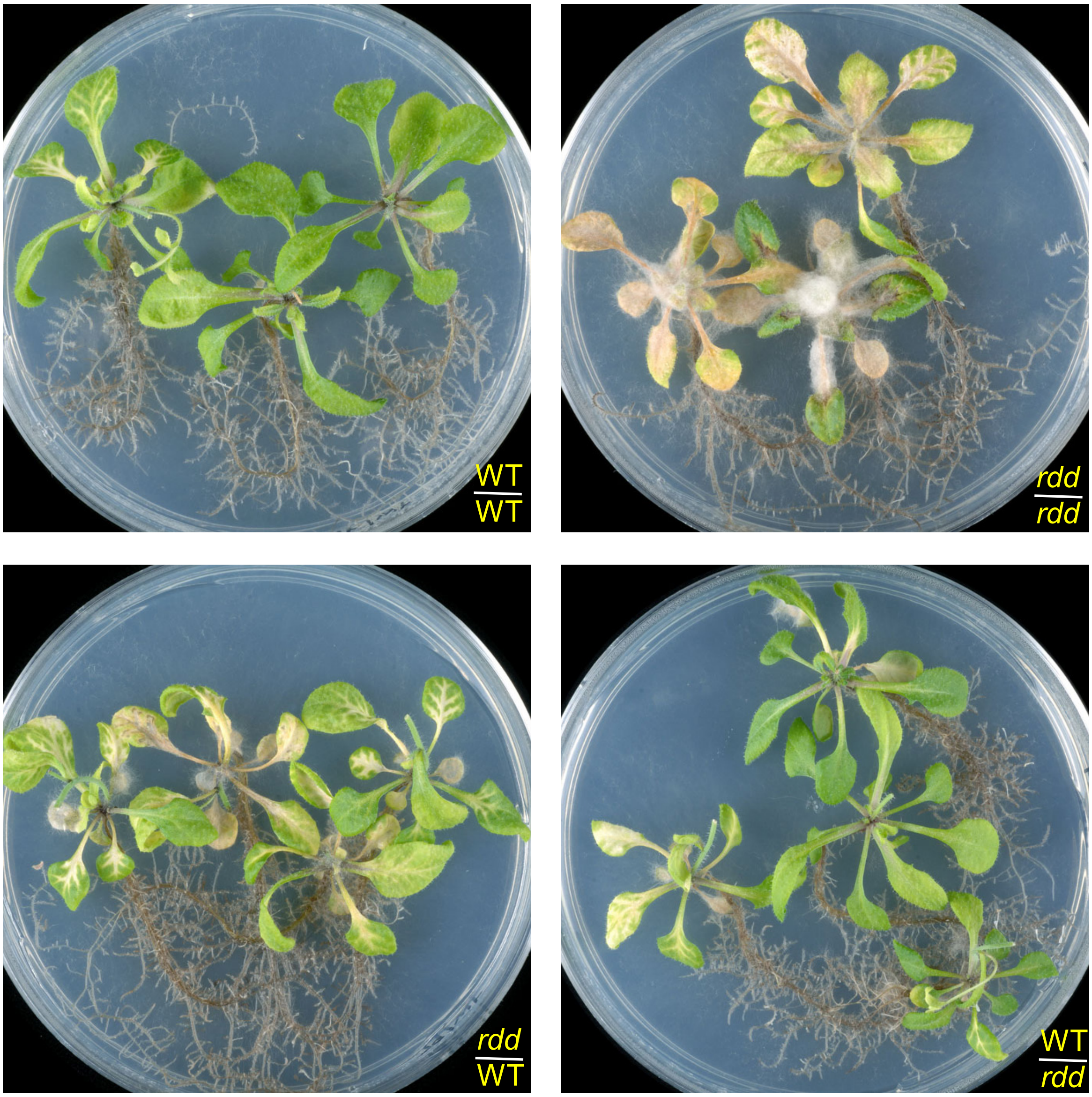

Figure S1. **Grafted plants between *rdd* and Col-0 show an intermediate disease phenotype in response to *Fo* infection**. Grafted plants were inoculated with *Fo* and grown at 22°C on MS[S-] plate . Photographs were taken at 14 dpi. The scion and rootstock of each graft are indicated by the labels above and below the line, respectively.

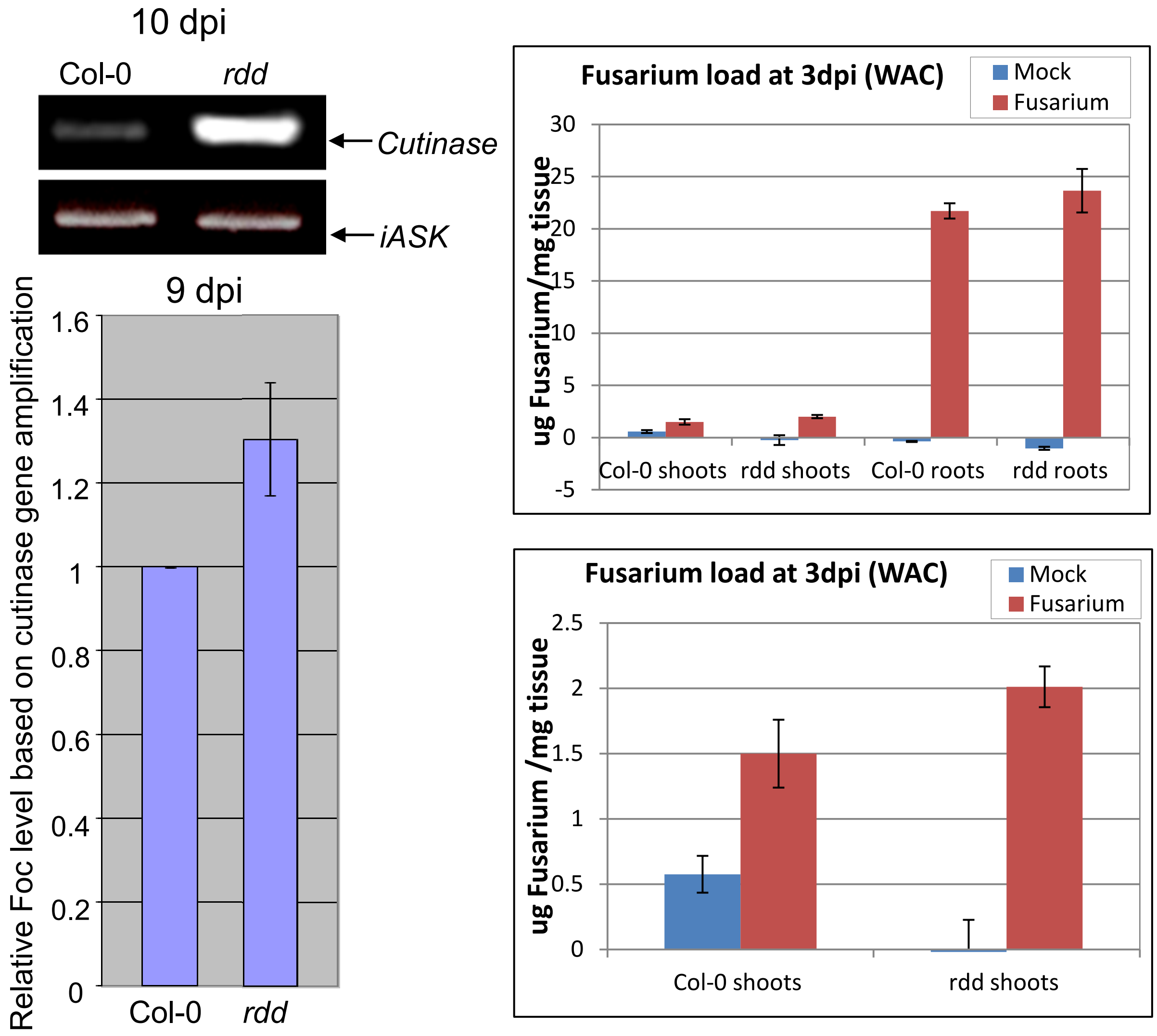

Figure S2. Estimation of *F. oxysporum* biomass in wild-type and *rdd* plants. Left: Fungal biomass was quantified in roots of wild-type and *rdd* mutant plants grown in soil by assaying *F. oxysporum*-specific gene Cutinase using semi-quantitative RT-PCR at 10 dpi (top) or RT-qPCR at 9 dpi (bottom) . The host Arabidopsis housekeeping gene *iASK* was amplified as loading control. **Right:** Fungal titre was measured in shoots and/or roots of Col-0 and *rdd* plants grown on MS[S-] plate using the florescence-chitin method (Ayliffe et al., A simple method for comparing fungal biomass in infected plant tissues. *Mol Plant Microbe Interact.* 2013 26:658-67). T-test shows significant difference between Col-0 and *rdd* in shoots (p=0.043) but not in roots.

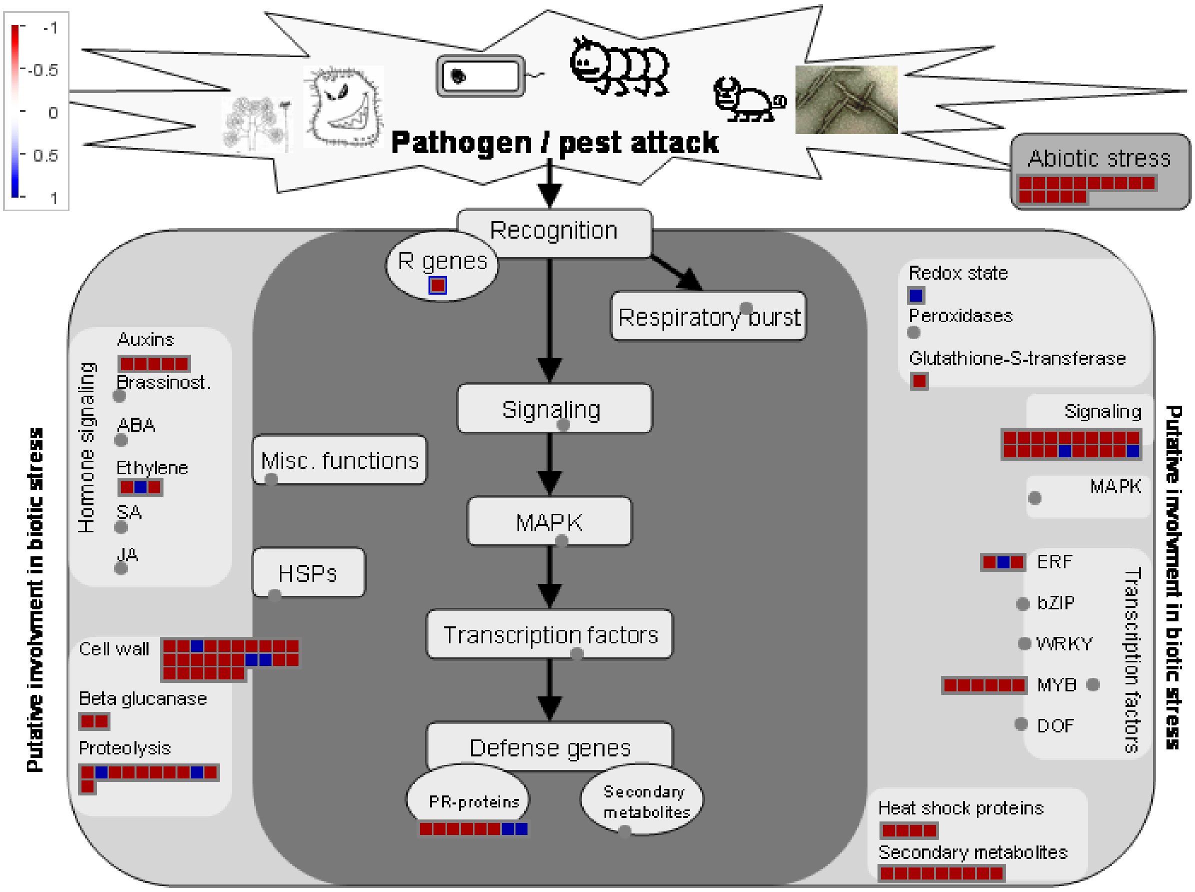

Figure S3. **MapMan analysis of the 348 genes differentially expressed between *rdd* and Col-0 identified 111 genes (representing 115 data points) as biotic stress response genes**. Each coloured square represents a gene that is either up-regulated (blue) or down-regulated (red) in the *rdd* mutant by ≥ 2-fold compared to Col-0. The scale on the top left represents the fold change in log_2 base. Most of these genes (99) are down-regulated in the *rdd* mutant.

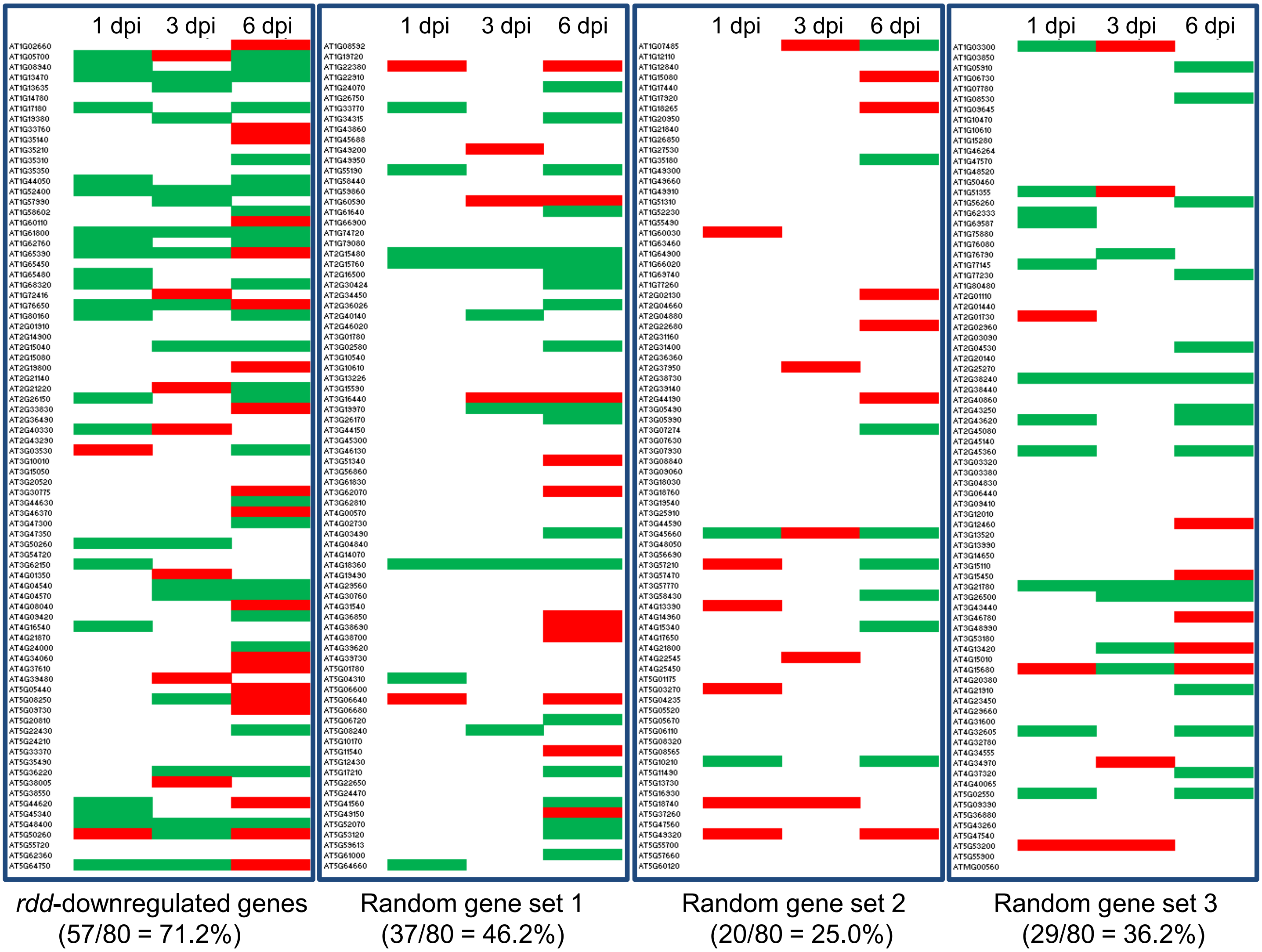

Figure S4A. The *rdd*-downregulated stress response genes are enriched for *Fo*-responsive expression pattern. Eighty of the 160 *rdd*-downregulated stress response genes shown in Table S5 have sequence reads in the RNA-seq data of *Fo*-infected and uninfected Col-0 plants at the three time points (1, 3, 6 dpi), of which 57 (72.5%) show ≥ 2 fold differential expression in *Fo*-infected Col-0 plants in comparison to uninfected plants at one or more of the three time points (left). Three sets of 80 randomly selected genes (all having sequence reads in the RNA-seq data) show no such enrichment for *Fo*-induced differential expression.

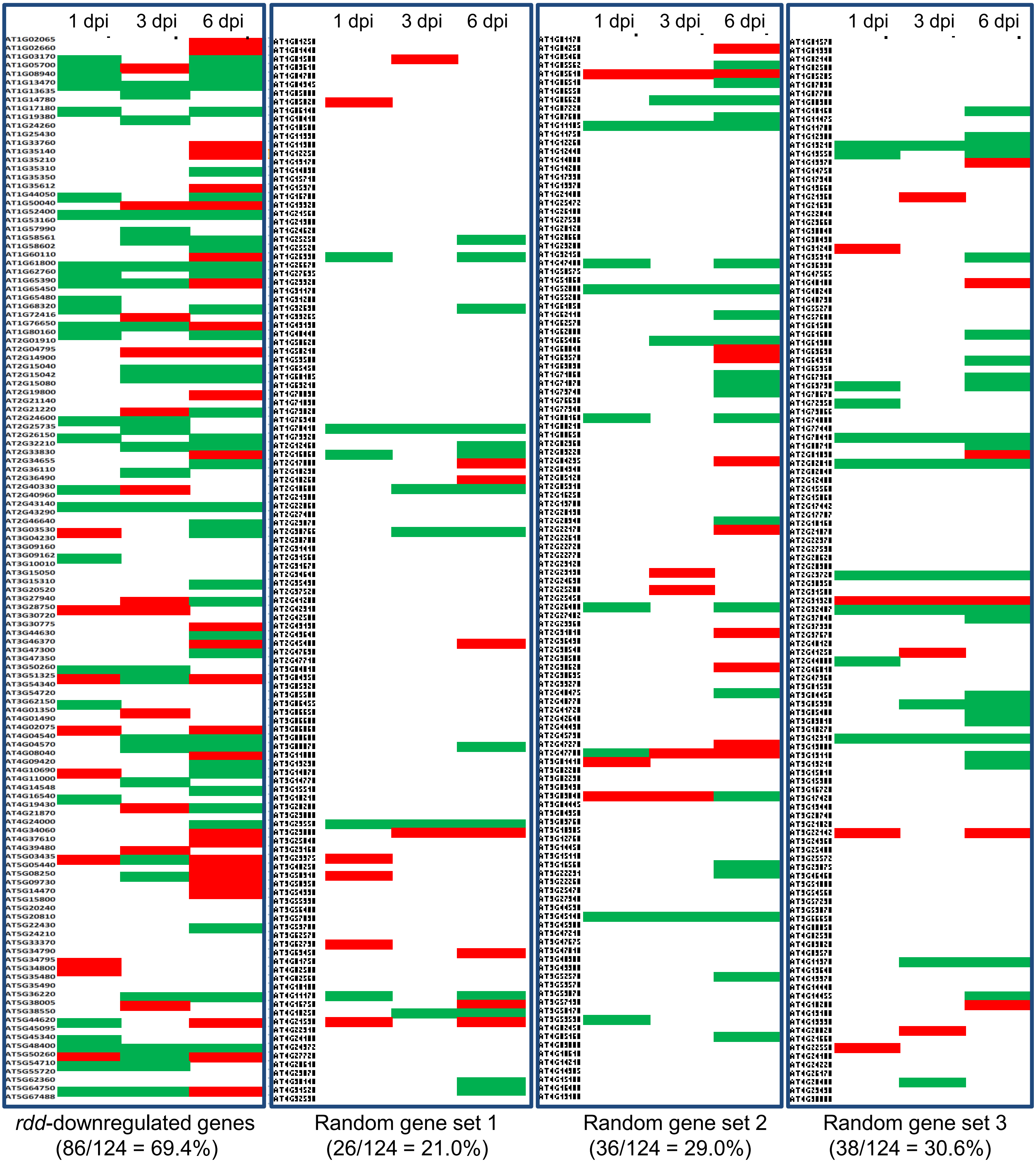

Figure S4B. **The *rdd*-downregulated genes are enriched for *Fo*-responsive expression pattern.** 124 of the 279 *rdd*-downregulated genes shown in Table S1 have sequence reads in the RNA-seq data of *Fo*-infected and uninfected Col-0 plants at the three time points (1, 3, 6 dpi), of which 86 (69.4%) show ≥ 2 fold differential expression in *Fo*-infected plants in comparison to uninfected plants at one or more of the three time points (left). Three sets of 124 randomly selected genes (all having sequence reads in the RNA-seq data) show no such enrichment for *Fo*-induced differential expression. Green lines, upregulation in the RNA-seq data; red lines, downregulation in the RNA-seq data.

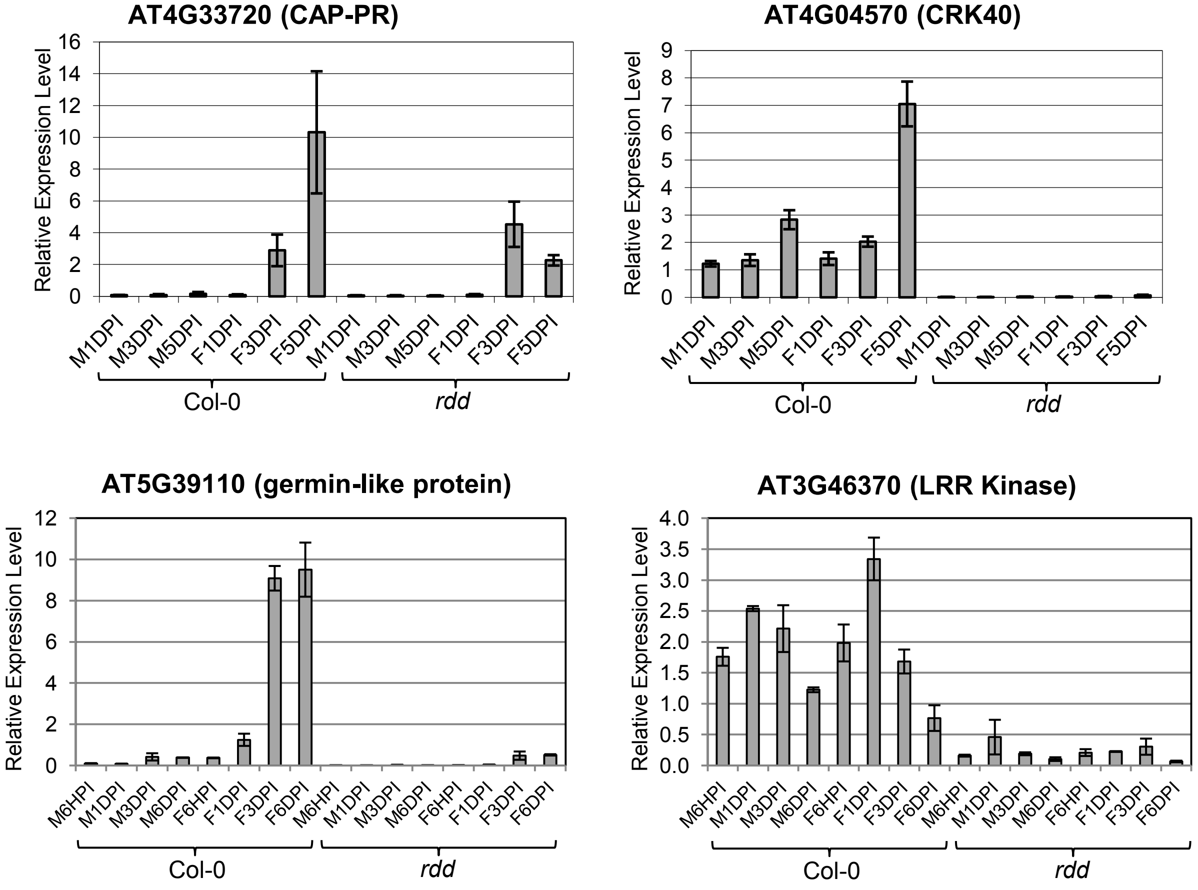

Figure S5. **Additional RT-qPCR analyses of the stress response genes *CAP-PR*, *LRR-kinase*, and *CRK40* genes**. The Actin 2 gene was used as the internal reference.

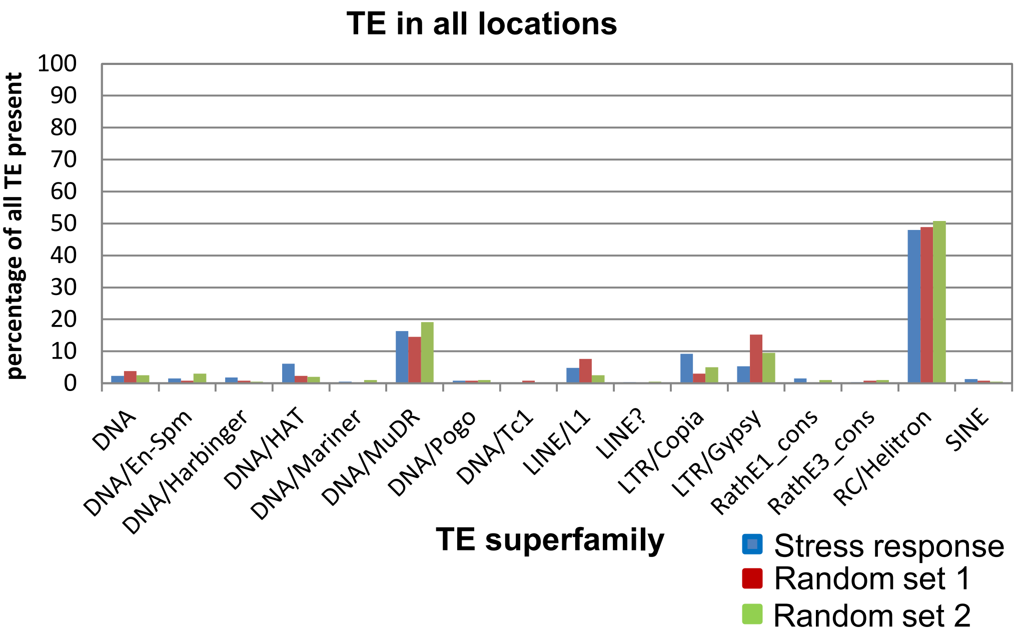

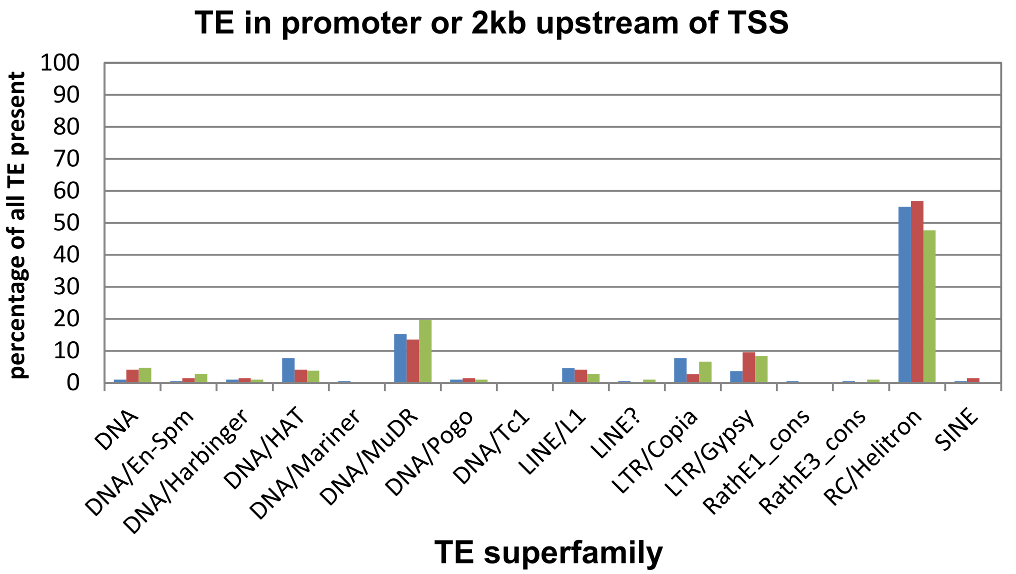

Figure S6. **Classification of TEs in *rdd*-regulated genes and randomly selected genes.** The Y-axis indicates the percentage of TE sequences in the total number of TE sequences identified in each of the three sets of genes, namely the *rdd*-downregulated stress response genes (blue), and two random sets of genes (red and green).

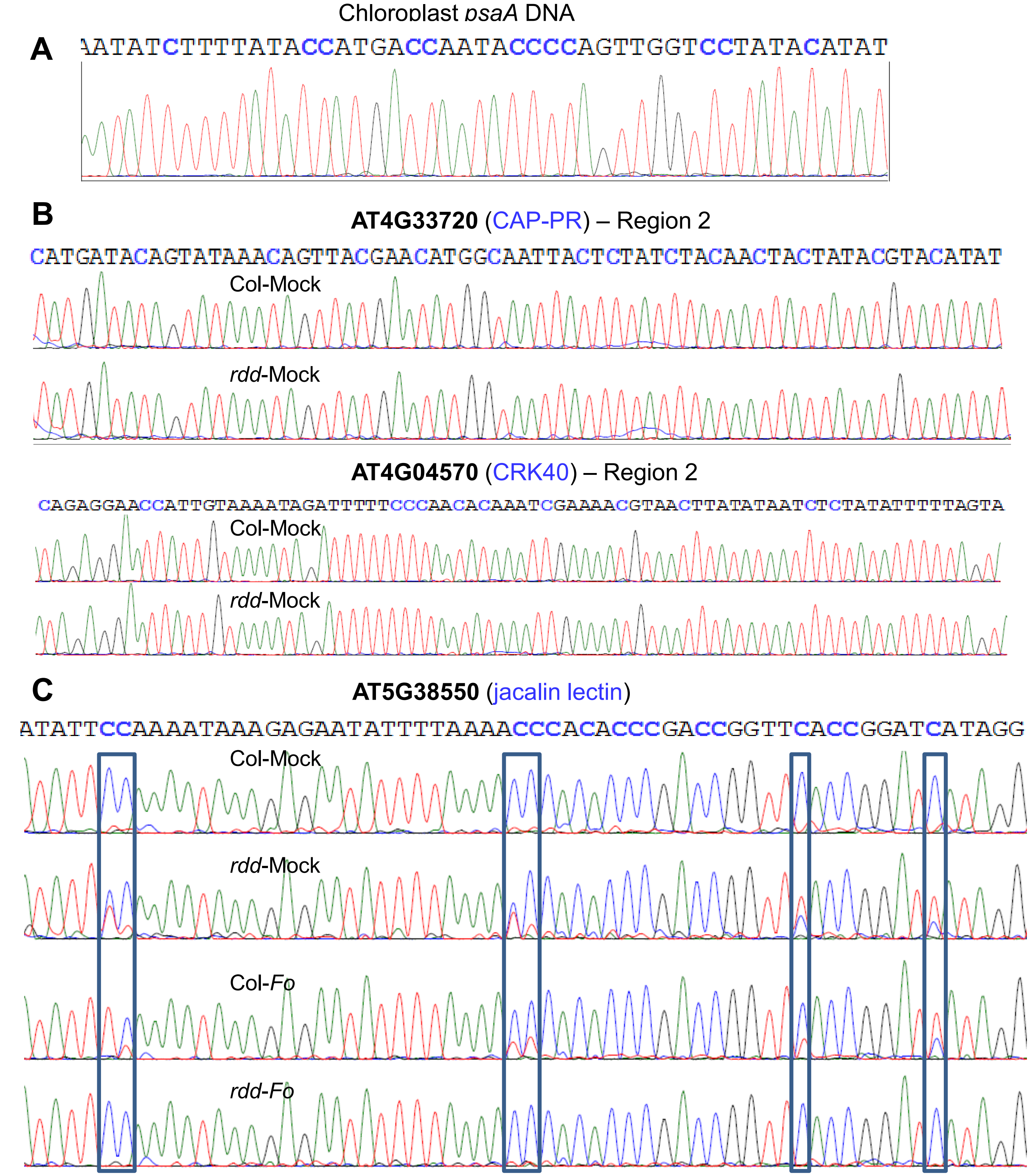

Figure S7. Examples of sequencing trace files of bisulfite PCR product. (A) Bisulfite conversion is efficient, as indicated by the lack of cytosines in the PCR product of the chloroplast *psaA* gene. All 24 samples look the same. (B) The regions of promoters distal to the TE sequences in AT4G33720 and AT4G04570 show no cytosine methylation in either Col or *rdd*. (C) Examples of differential cytosine methylation in AT5G38550 between Col and *rdd*, and between mock-treated and *Fo*-infected plants. Note the difference in the proportion of cytosine peaks (blue) versus thymine peaks (red) between the different samples.

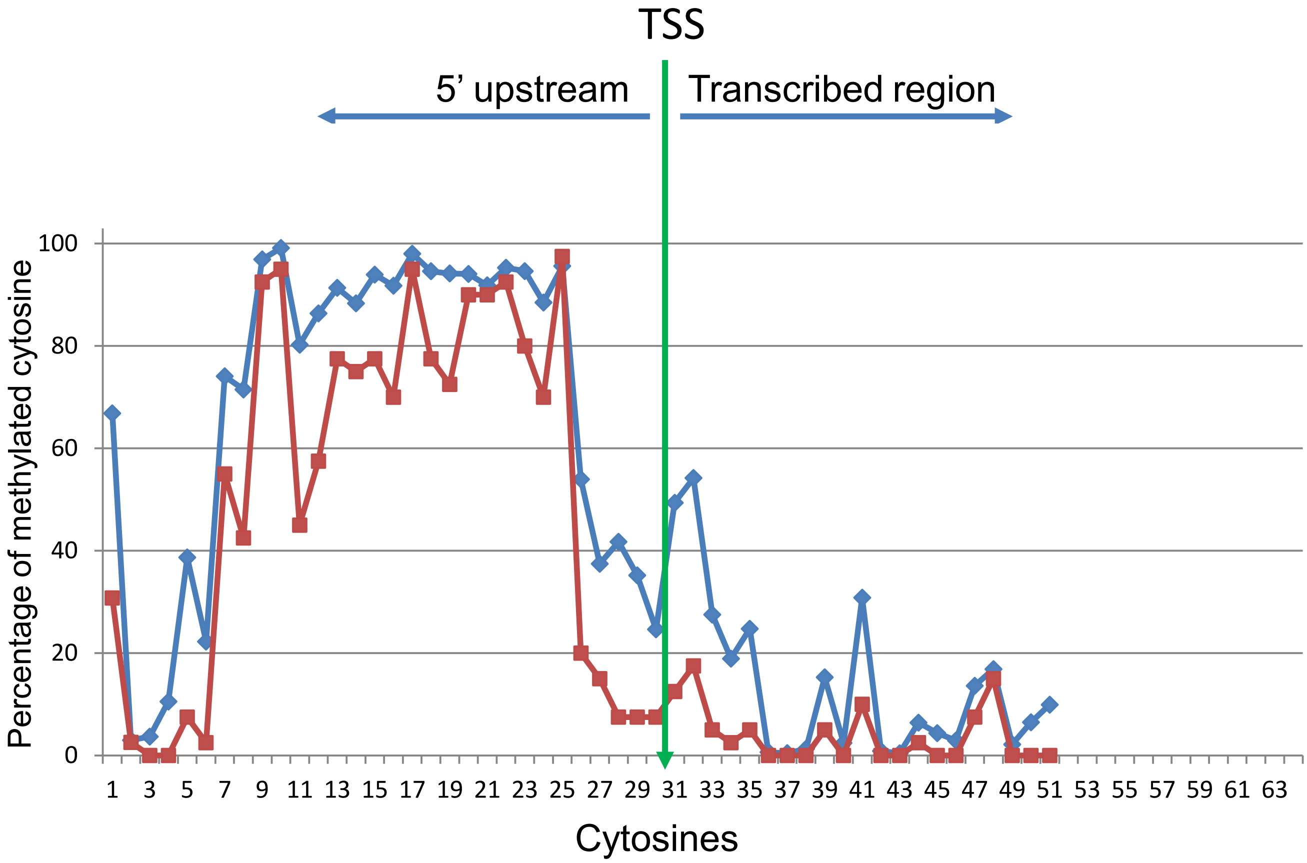

Figure S8. **Methylation levels in Region 2 of AT1G58602 in *Fo*-infected *rdd* (1 dpi) determined by direct sequencing of PCR product (blue line) and by sequencing of 40 pGEM T Easy clones of the PCR product (red lines).** Cytosines in all sequence contexts, including CG, CHG and CHH, are included according their natural order of occurrence in the sequence. The blue line is smoother than the red line, suggesting that results from direct sequencing of PCR product is more reliable than from sequencing of individual PCR clones. Also, among the 40 pGEM T Easy clones sequenced, 27 (68%) had insert in one orientation and 13 (32%) in the other orientation, suggesting that different PCR sequences are not evenly cloned.

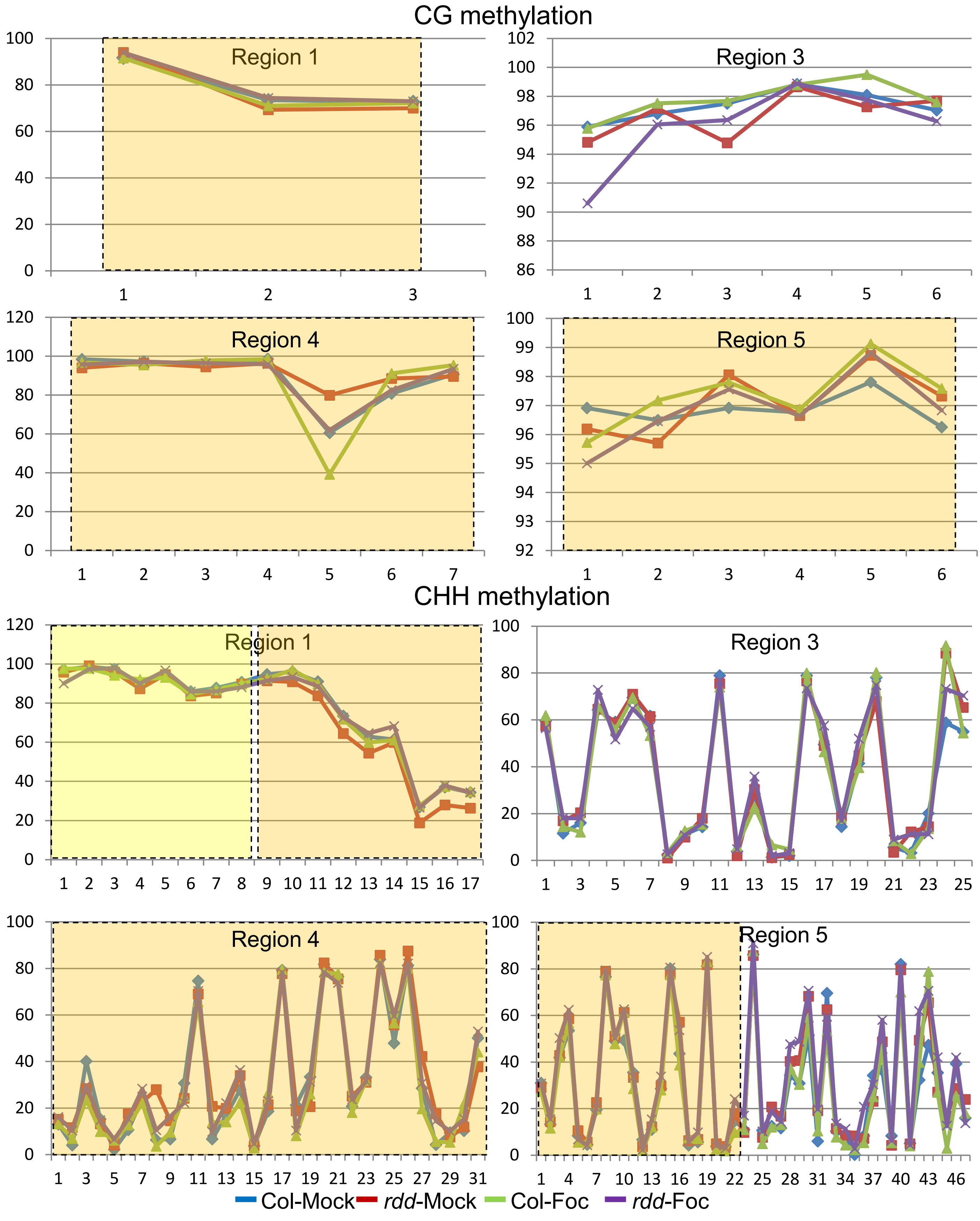

Figure S9. **Sequences in Regions 1, 3, 4 and 5 of AT1G58602 show no clear difference in methylation between *rdd* and Col.** X-axis represent cytosines along the sequenced region in their natural order, and Y-axis shows the percentage of methylated cytosines. Regions overlapping with TEs are highlighted with yellow or orange shades.

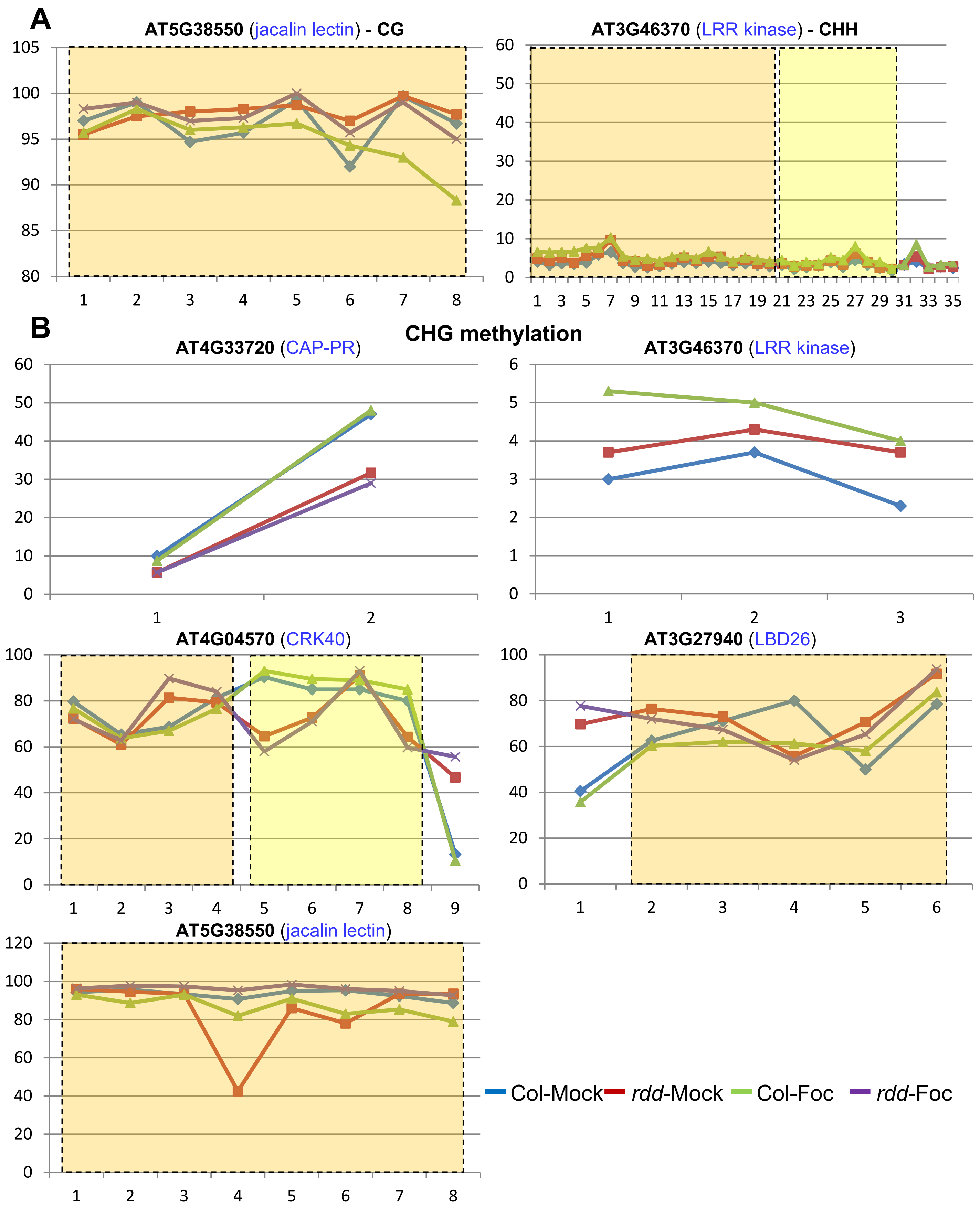

Figure S10. **Additional bisulphite sequencing results showing (A) similar levels of CG methylation (AT5G38550), absence of CHH methylation (AT4G33720) or (B) lack of a clear pattern for CHG methylation.** Regions overlapping with TEs are highlighted with yellow or orange shades.

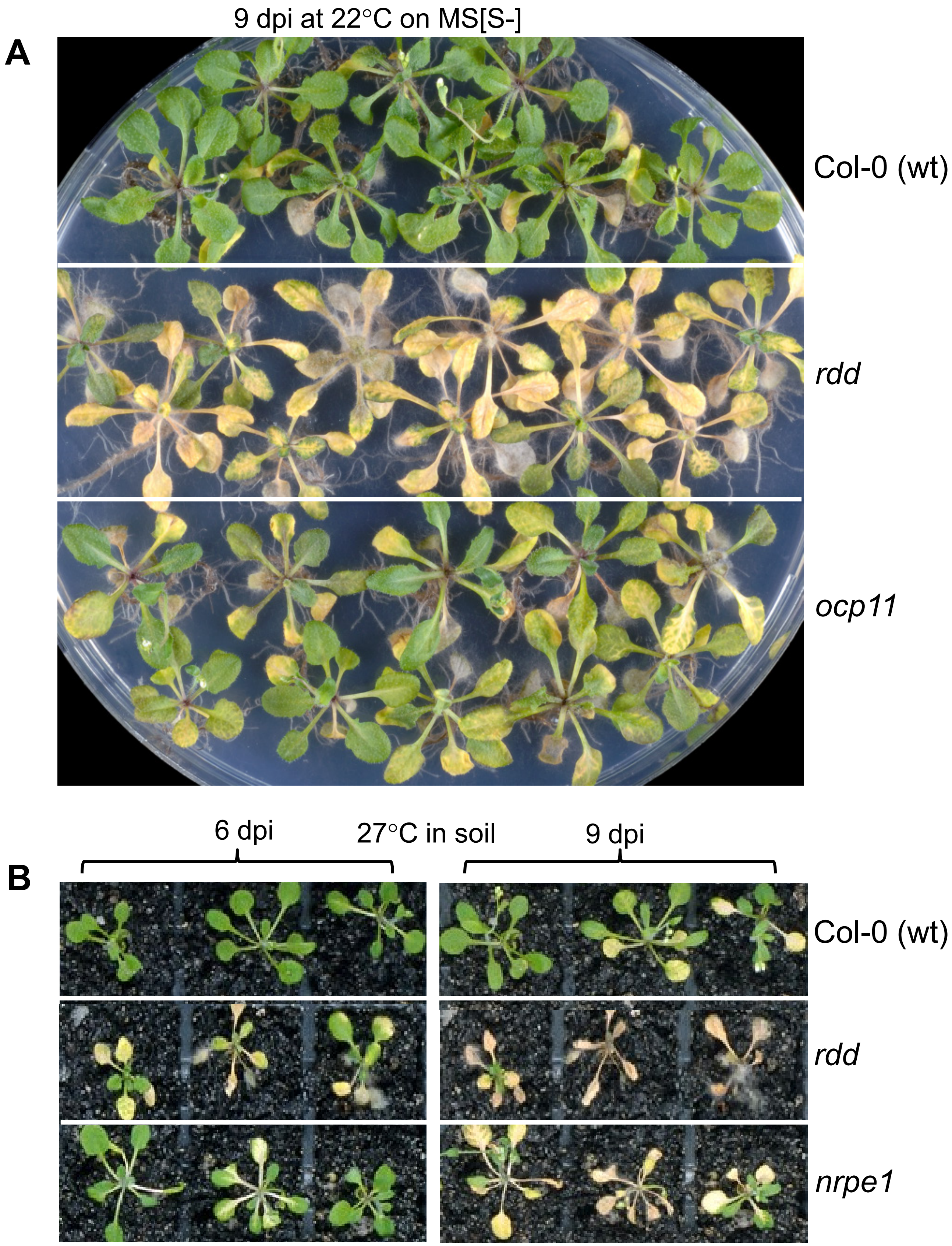

Figure S11. **The RdDM mutant *ocp11* (*ago4*) and *nrpe1* (*pol v*) are also susceptible to *Fo* infection but to a lesser degree than *rdd*.**

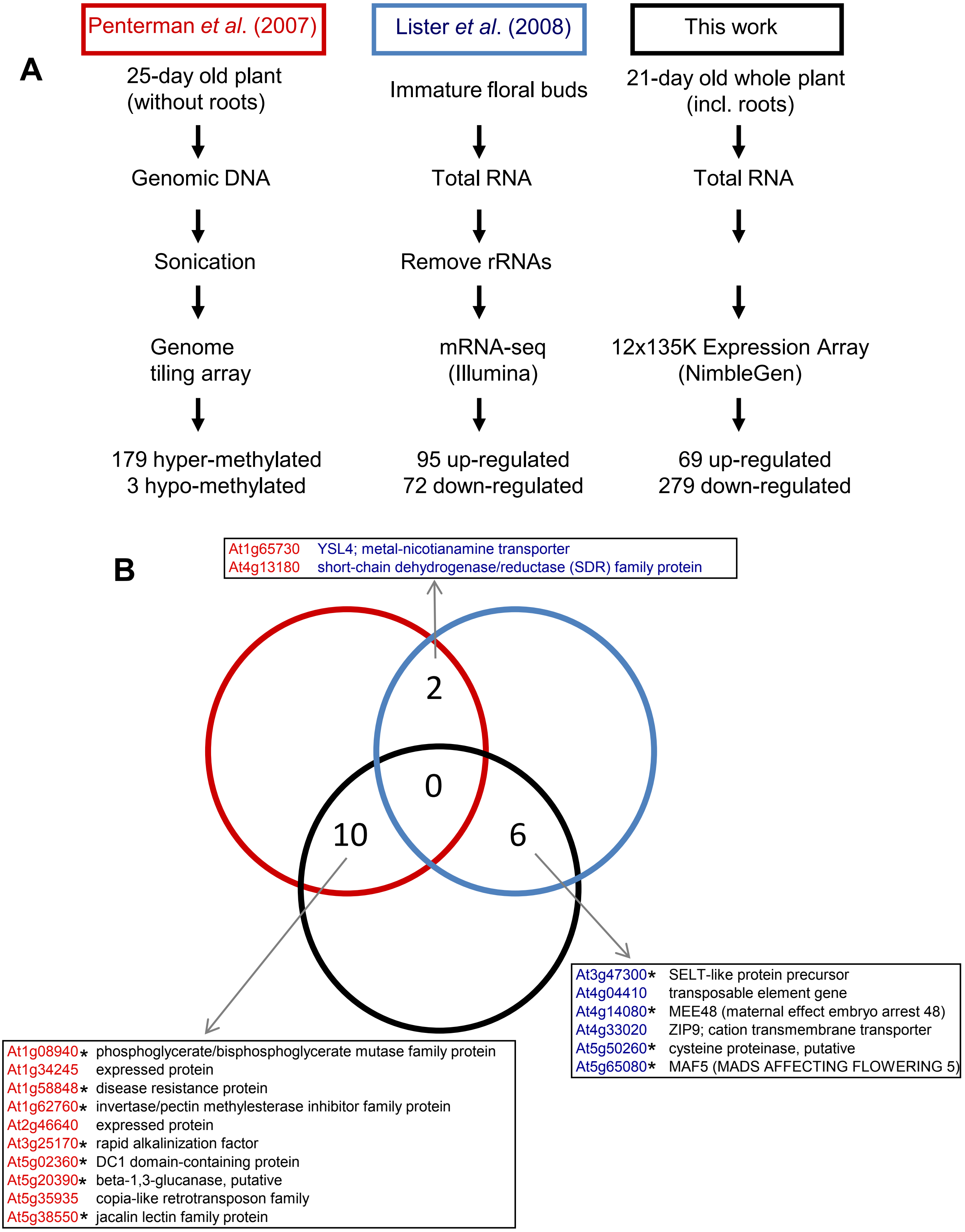

Figure S12. Differentially methylated or expressed genes in *rdd* from three independent studies show only limited overlap. (A) Plant materials and techniques used in the three studies. (B) Venn diagram showing the number of overlapped genes among the three studies. The details of these genes are given on the side. Asterisks indicate genes present in the full stress response gene list in Table S5.

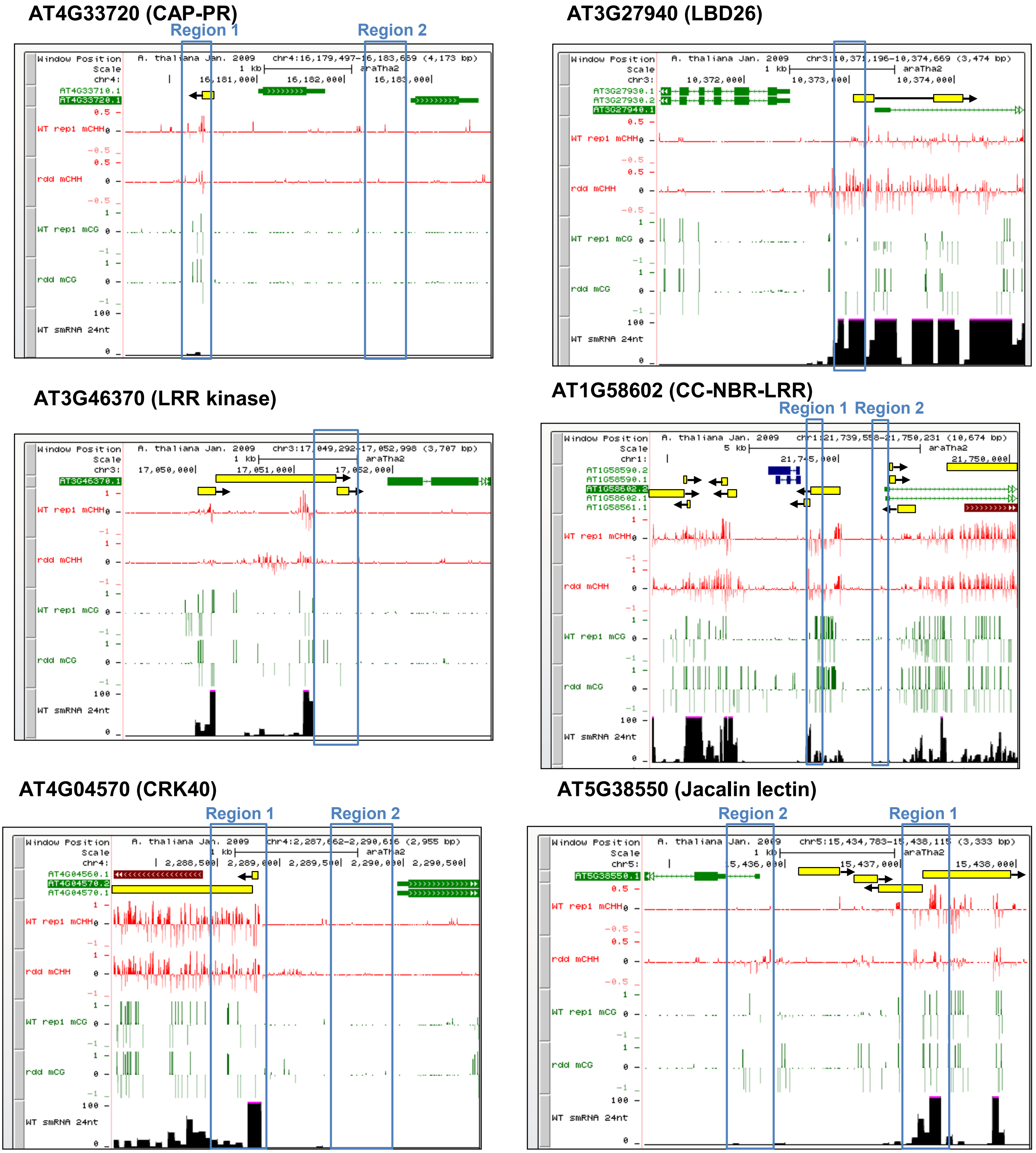

Figure 13A. Genome browser (Stroud et al 2013; Ref. #26) screen shots of the six bisulfite-sequenced stress response genes analysed in this current study. Shown are regions upstream of the start of the genes of interest (shown as green bars with gene name highlighted in green on the left). The bisulfite-sequenced regions in this study are indicated with a blue box and TE elements are shown in yellow. Consistent with this current study, i) Regions 2 of AT4G33720 and AT4G04570, which are distal to TE, show little or no methylation; ii) Region 2 of AT5G38550 shows induction of methylation in both CHH and CG contexts in *rdd*; iii) the region in AT3G46370 shows induced CG methylation in *rdd*; iv) Region 1 of AT4G33720 shows hypo-^{m}CHH but increased CG methylation; v) Regions 1 of AT4G04570 and AT5G38550 both show hypo-^{m}CHH in *rdd* although no clear change in CG methylation due to high levels in both Wt and *rdd* plants; and vi) the region in AT3G27940 shows increased CG methylation in *rdd*, although this region also shows increased CHH methylation, which is different to the slight reduction in CHH methylation of this study. Another exception is Region 2 of AT1G58602, which shows very little methylation in both WT and *rdd* plants, in contrast to this study showing strong gain of methylation in *rdd*. Changes in Pol IV and Pol V mutants across these regions are noted in Supplementary Table S9. Note that most of the regions showing changes in methylation either overlap or are adjacent to 24-nt siRNA clusters.

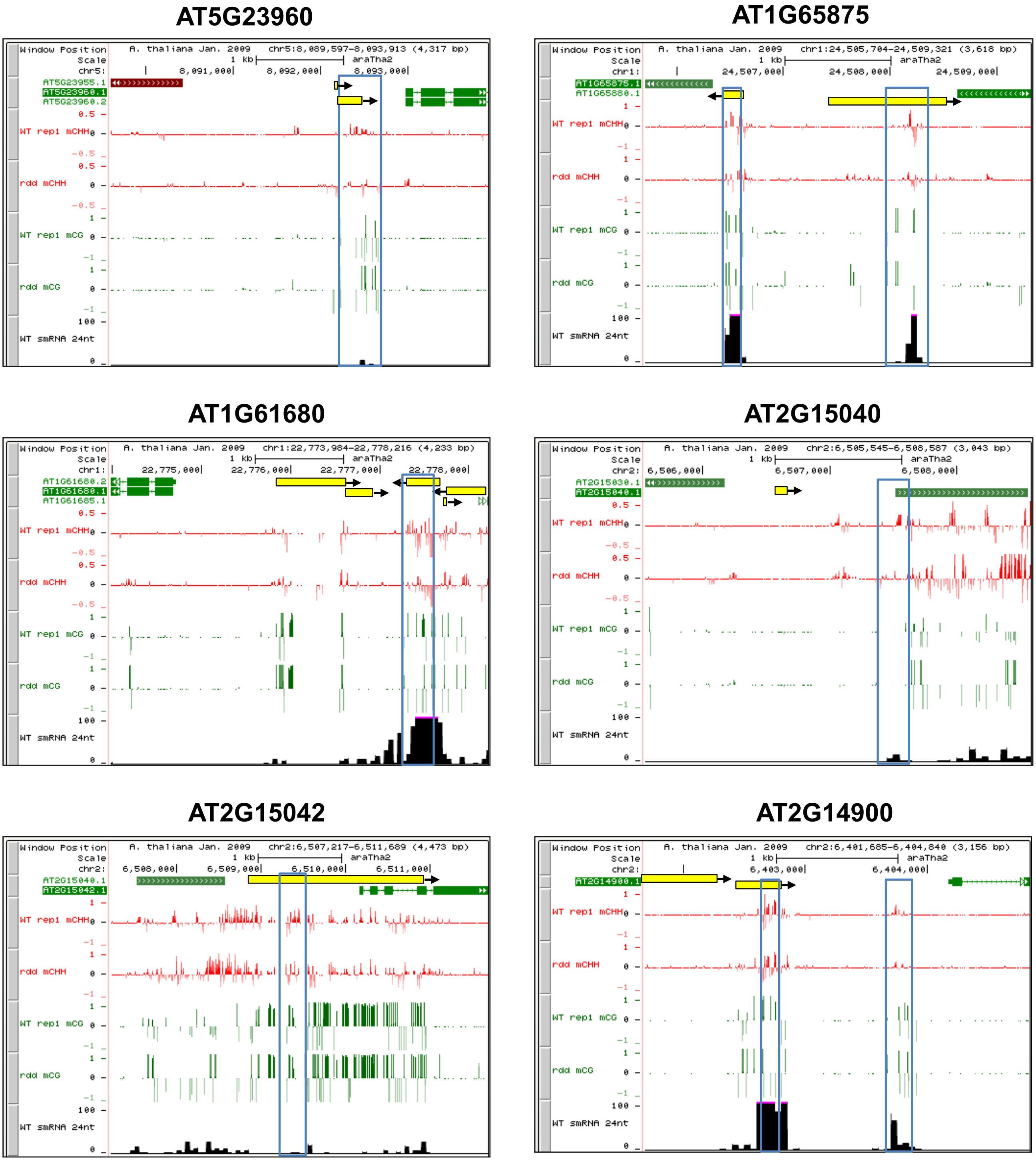

Figure 13B: Genome browser (Stroud *et al*. 2013; Ref. #26) screen shots of 11 additional genes (continued next page) showing short regions of hypo-^{m}CHH (blue box). Most of these regions coincide with TE elements (shown in yellow) and small RNA target regions (Lee *et al.* 2012; Ref. #29). These 11 genes are highlighted in red in Supplementary Table S9.

AT5G38005

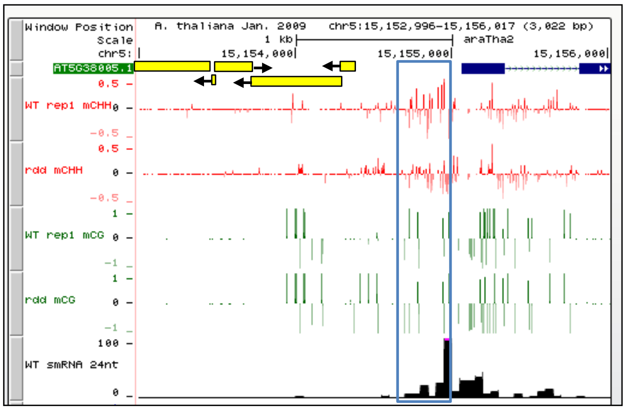

AT5G35490

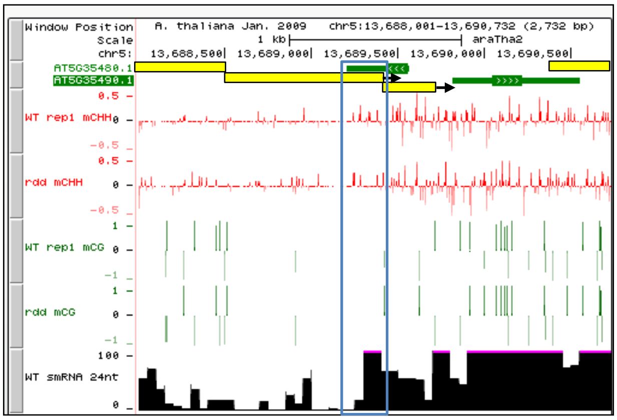

AT1G35140

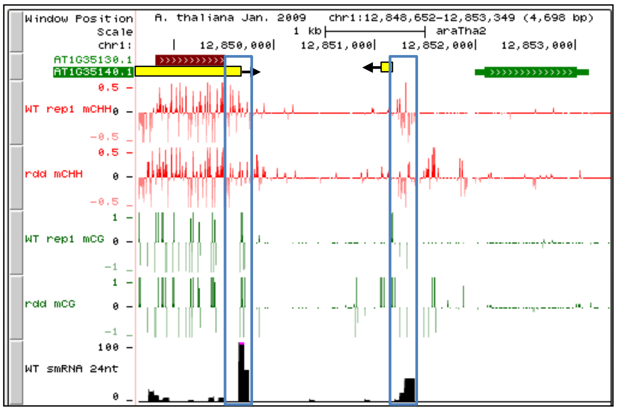

AT4G07820

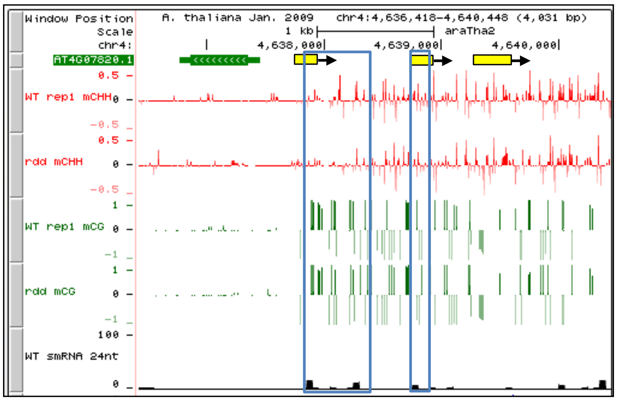

AT1G24520

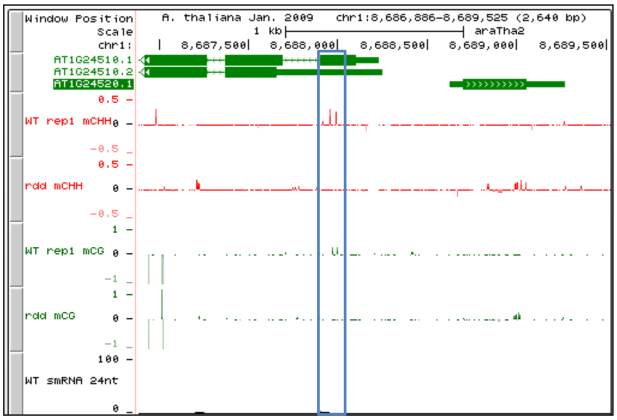

Figure S13B - continued
